# Unraveling the complex interplay: immunopathology and immune evasion strategies of alphaviruses with emphasis on neurological implications

**DOI:** 10.3389/fcimb.2024.1421571

**Published:** 2024-08-15

**Authors:** Raquel de Oliveira Souza, José Wandilson Barboza Duarte Júnior, Victória Simões Della Casa, Daniela Santoro Rosa, Laurent Renia, Carla Claser

**Affiliations:** ^1^ Department of Parasitology, Institute of Biomedical Sciences, University of São Paulo (USP), São Paulo, Brazil; ^2^ Department of Microbiology, Immunology and Parasitology, Federal University of São Paulo (UNIFESP), São Paulo, Brazil; ^3^ ASTAR Infectious Diseases Labs (ASTAR ID Labs), Agency for Science, Technology and Research (ASTAR), Singapore, Singapore; ^4^ Lee Kong Chian School of Medicine, Nanyang Technological University, Singapore, Singapore; ^5^ School of Biological Sciences, Nanyang Technological University, Singapore, Singapore

**Keywords:** alphavirus, chikungunya, mayaro, mouse, coinfection, nervous system, immunopathology, immune evasion

## Abstract

Arthritogenic alphaviruses pose a significant public health concern due to their ability to cause joint inflammation, with emerging evidence of potential neurological consequences. In this review, we examine the immunopathology and immune evasion strategies employed by these viruses, highlighting their complex mechanisms of pathogenesis and neurological implications. We delve into how these viruses manipulate host immune responses, modulate inflammatory pathways, and potentially establish persistent infections. Further, we explore their ability to breach the blood-brain barrier, triggering neurological complications, and how co-infections exacerbate neurological outcomes. This review synthesizes current research to provide a comprehensive overview of the immunopathological mechanisms driving arthritogenic alphavirus infections and their impact on neurological health. By highlighting knowledge gaps, it underscores the need for research to unravel the complexities of virus-host interactions. This deeper understanding is crucial for developing targeted therapies to address both joint and neurological manifestations of these infections.

## Introduction

Alphaviruses represent a broad group of medically and economically important viruses with the potential for epidemic outbreaks. These enveloped viruses, members of the Togaviridae family, possess a single-stranded, positive-sense RNA genome (Strauss and Strauss, 1994). Despite low mortality rates, alphaviruses cause significant morbidity in humans worldwide, and different species display distinct geographical distributions. For example, Chikungunya has impacted Oceania, Asia, Africa, and, in the last decade, the Americas (Morens and Fauci, 2012). Climate change and travel have intensified the risk of new alphavirus disease outbreaks (Bellone et al., 2023).

Alphaviruses are primarily transmitted by arthropod vectors, with mosquitoes as the main culprits. Several mosquito species of the *Aedes, Culex*, and *Anopheles* genera are involved, although in some cases, other arthropod species may play a role. Alphaviruses frequently maintain transmission cycles between mosquitoes and various vertebrate hosts, such as birds, rodents, or other mammals. Humans generally act as incidental or “dead-end” hosts (Schmaljohn and McClain, 1996).

The pathologies resulting from alphavirus infections in humans vary widely. While many alphaviruses cause only mild or asymptomatic infections, some can lead to severe illnesses. Human-infecting alphaviruses can be broadly classified into two groups. The first group includes arthritogenic alphaviruses such as Chikungunya virus (CHIKV), Mayaro virus (MAYV), O’nyong’ nyong virus (ONNV), and Ross River virus (RRV) that are responsible for debilitating fever, joint pain (polyarthralgia and polyarthritis), and sometimes rash (trunk, limbs and face) (Zaid et al., 2021). The second group includes encephalitic alphaviruses like eastern equine encephalitis virus (EEEV), venezuelan equine encephalitis virus (VEEV), and western equine encephalitis virus (WEEV) that can cause severe fever, meningitis, and encephalitis (Zacks and Paessler, 2010). While primarily associated with joint or muscle pain (arthritogenic) or brain inflammation (encephalitic), alphaviruses can sometimes disseminate into the nervous system in other ways, causing atypical encephalitis, Guillain-Barré Syndrome or other neurological impairments (sensory issues, paralysis, and chronic neurological problems) (Baxter and Heise, 2020).

While vaccines exist and have been licensed for some alphaviruses (Ng and Renia, 2024), there is currently no specific treatment or cure for alphavirus infections. However, different therapeutics approaches are being investigated: i) small molecules such as nucleoside/nucleotide analogues, or compounds targeting enzymes involved in viral replication (like proteases or polymerases) (Battisti et al., 2021); ii) Interferon-based interventions. Interferons are potent effector molecules with strong antiviral activities. Administration of interferons or stimulating their production is an avenue to generate or boost the antiviral response (Lazear et al., 2019); iii) host-directed therapies targeting inflammation. The severe symptoms of many alphaviral diseases (arthritis, encephalitis) stem from excessive inflammation. Therapies that can modulate specific inflammatory pathways are being explored to decrease tissue damage and pain (Mostafavi et al., 2019); and iv) antibody-based therapies. Two approaches have been pursued. Monoclonal Antibodies that neutralize alphaviruses have been identified. They target viral surface proteins, preventing virus entry into cells or helping in virus clearance through their Fc receptors (Kim and Diamond, 2023). Another approach using polyclonal IgG purified from convalescent plasma has been investigated and was shown to protect mice against chikungunya (Couderc et al., 2009). It remains to be shown if this treatment will also be effective against human infections. In the future, combined therapies that target different stages of the virus life cycle, can be taken preventatively by high-risk populations or during outbreaks. They may be more effective and hinder resistance development.

Alphaviruses, like many pathogens, employ various strategies to evade or manipulate the immune system. One such tactic involves blocking the interferon signaling pathways, which are crucial components of the body’s initial defense against viruses. By suppressing interferon production and disrupting its signaling pathways, alphaviruses facilitate their replication (Schoggins et al., 2011). Additionally, certain alphaviruses can establish persistent reservoirs in tissue cells, evading immune detection and potentially leading to longer-term disease (Ryman and Klimstra, 2008). Furthermore, alphaviruses can disrupt the functions of various immune cells, impairing their ability to eliminate the virus and setting conditions for persistent and/or chronic damages (Trobaugh and Klimstra, 2017).

## Host immune response against alphavirus

A comprehensive understanding of host immune responses during alphavirus infection is crucial for the development of targeted antiviral and immunotherapeutic strategies and effective vaccines. While the innate immune response acts as the first line of defense, the adaptive immune system plays a crucial role in controlling and eliminating the virus (during acute infection). Importantly, both innate and adaptive immune responses contribute to protective and potentially harmful mechanisms.

Studies in humans and animal models demonstrate that the pathogenesis of arthritogenic alphaviruses is linked to immune cell infiltration (including NK cells, monocytes/macrophages, and T cells) into affected joints, along with elevated levels of specific cytokines (Manimunda et al., 2010). However, the precise mechanisms driving alphavirus-induced joint disease still require further clarification.

Here, we explore recent advancements in our understanding of innate and adaptive immune responses elicited during alphavirus infections. Insights into the interplay between immune cells, the dynamics of immunological memory, and the factors influencing long-term protection pave the way for targeted interventions against these important viruses.

### Innate immune responses

Overall, the innate immune response to alphavirus infection involves several key steps: recognition of viral components by pattern recognition receptors, production of interferons and pro-inflammatory cytokines, and the activation and recruitment of immune cells. By unraveling the complexities of these innate immune responses during acute alphavirus infection, we can develop innovative therapeutic approaches to better control and mitigate the impact of these viruses.

### Recognition of alphaviral pathogen-associated molecular patterns

**Figure 1:**
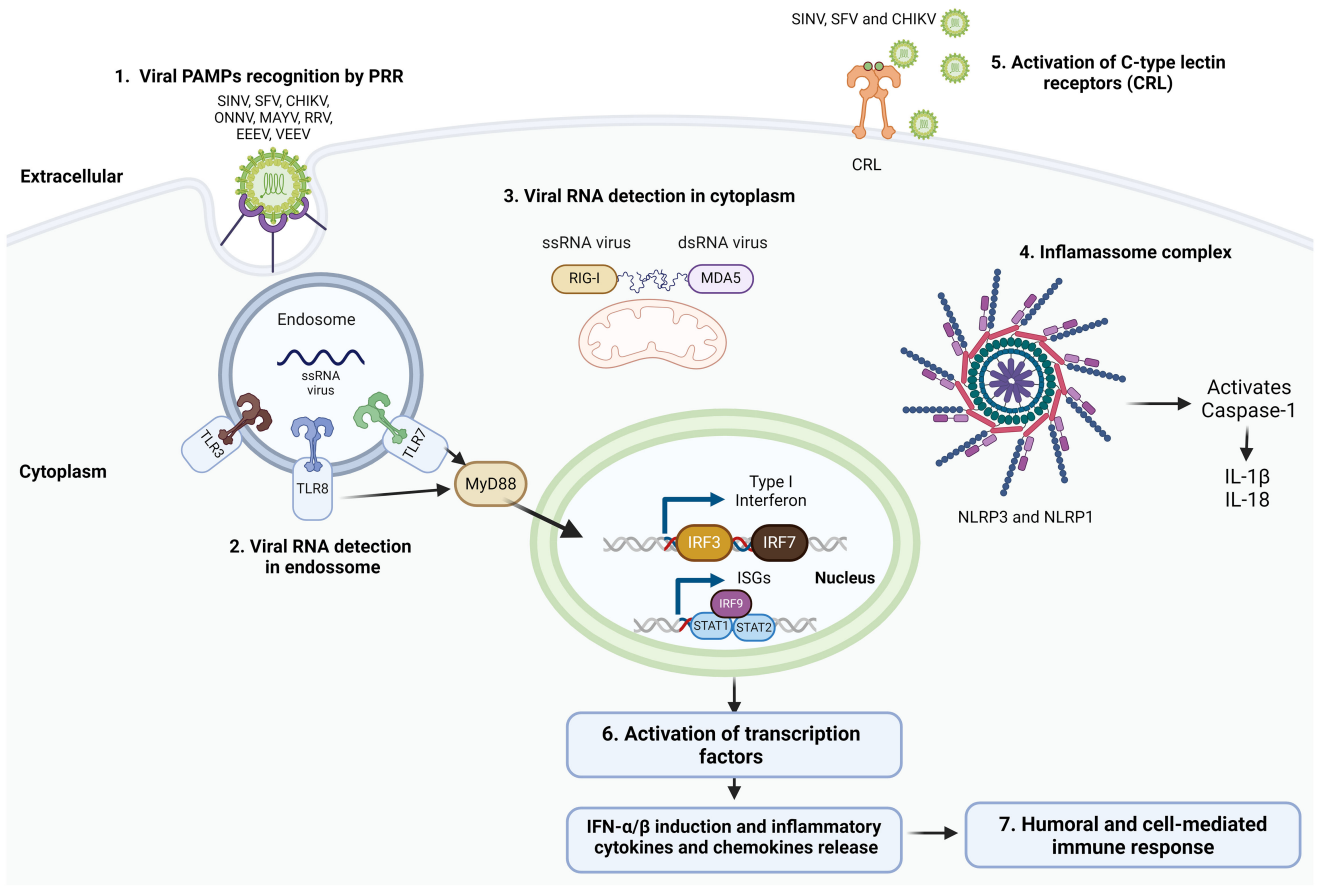
Alphavirus induced innate immune response. The innate immune response induced by alphaviruses involves a series of key mechanisms. (1) Alphavirus recognition is mediated by pattern recognition receptors (PRRs) that detect pathogen-associated molecular patterns (PAMPs) associated with the invading viruses. This recognition is facilitated by various receptors, including (2) TLR3, TLR7, and TLR8 located in endosomes, as well as (3) RIG-I-like receptors (RLRs) such as RIG-I and MDA5 found in the cytoplasm. These receptors detect viral RNA and initiate antiviral responses by activating signaling cascades that ultimately lead to the activation of transcription factors. (4) NOD-like receptors (NLRs), including NLRP3 and NLRP1, form inflammasomes that activate caspase-1, resulting in the production of pro-inflammatory cytokines IL-1β and IL-18. (5) C-type lectin receptors (CLRs) are also activated during alphavirus infection, playing a role in modulating the immune response. (6) The activation of PRRs triggers signaling cascades that culminate in the activation of transcription factors. This activation leads to the expression of type I interferons (IFNs), IFN-stimulated genes (ISGs), as well as the production of pro-inflammatory cytokines and chemokines. (7) These responses are crucial for mounting an effective antiviral humoral and cell-mediated immune response against alphaviruses. [Created with BioRender.com with license no. OL270LDGL6.].

Our immune system fights off infections by recognizing conserved patterns of viruses called pathogen-associated molecular patterns (PAMPs). Alphaviruses, in particular, can be detected by several sensors within our cells, known as pattern recognition receptors (PRRs). including Toll-like receptors (TLRs), retinoic acid-inducible gene I (RIG-I)-like receptors (RLRs), NOD-like receptors (NLRs), and C-type lectin receptors (CLRs) (Carpentier and Morrison, 2018; Kafai et al., 2022). TLRs and RLRs are key players in sensing viral RNA. TLRs (TLR3, TLR7, and TLR8) recognize viral RNA in endosomes, while RLRs such as RIG-I and melanoma differentiation-associated protein 5 (MDA5), sense viral RNA within the cytoplasm (Schlee and Hartmann, 2016). The activation of additional receptors, including NLRs and CLRs, has recently been described during alphavirus infection (Long et al., 2013; Chen and Ichinohe, 2015). NLRs, such as NLRP3 and NLRP1, form multiprotein complexes called inflammasomes, which activate caspase-1 and induce the production of pro-inflammatory cytokines IL-1β and IL-18. Recent studies have implicated NLRP3 and NLRP1 in the recognition of alphaviral PAMPs, suggesting their involvement in the induction of inflammatory responses during alphavirus infection (Jenster et al., 2023). Engagement of PRRs activates signaling cascades leading to the expression of transcription factors which ultimately induce type I interferons (IFNs), pro-inflammatory cytokines and chemokines (Carpentier and Morrison, 2018) (summarized in **Figure 1**).

### Role of type I IFN and inflammatory cytokines

The induction and secretion of IFNs, specifically IFN-α and IFN-β, are hallmarks of the innate immune response against viruses (Katze et al., 2002; Hertzog et al., 2011; Murira and Lamarre, 2016). Alphavirus-infected cells release IFNs, which acts in both autocrine and paracrine manners to establish an antiviral state by inducing the expression of interferon-stimulated genes (ISGs) that collectively contribute to inhibit viral replication and dissemination (Schoggins, 2019). Recent research has focused on the kinetics and dynamics of IFNs production during the early stages of alphavirus infection). The balance between IFNs induction and the virus’s ability to develop further rounds of infection is determined during the first few hours of virus replication, particularly when low numbers of cells and infectious virus are involved (Frolov et al., 2012). Alphaviruses, however, have evolved mechanisms to evade or antagonize the IFN response, thereby enhancing their ability to replicate and spread within the host (Liu et al., 2022b).

High levels of plasma IFN-α occurs during acute CHIKV infection in both humans and animal models (Ryman and Klimstra, 2008; Wauquier et al., 2011; Simarmata et al., 2016) and mice deficient in IFNs signaling have been shown to succumb to arthritogenic alphavirus infection (Ryman et al., 2000; Couderc et al., 2008; Ziegler et al., 2008; Schilte et al., 2010; Gardner et al., 2012; Seymour et al., 2013). Interestingly, IFN-α and IFN-β play different roles in protection; while IFN-α is important for limiting viral replication and spread, IFN-β limits neutrophil-mediated inflammation (Cook et al., 2019).

In addition to the IFN response, the production of inflammatory cytokines, chemokine and growth factors is a crucial aspect of the host immune response during alphavirus infections. Pro-inflammatory cytokines, such as interleukin-1β (IL-1β) and interleukin-6 (IL-6), play pivotal roles in orchestrating the inflammatory milieu. Several studies were carried out to characterize the cytokine profile and inflammatory mediators in patients with CHIKV. Differences in cytokine profile have been observed between the acute and chronic forms of the disease. In the acute phase, it is possible to observe high levels of IL-1β, IL-6, IL-7, IL-8, IL-12 and IL-15, which correlate with a higher viral load and more severe clinical manifestations (Goupil and Mores, 2016). In children, early viral clearance is associated with a higher production of IL-12p40, IL-1α, TNF-β, GM-CSF, and IFN-y (Simarmata et al., 2016).

Dysregulated inflammatory responses can contribute to immunopathology and severe disease outcomes. In patients with severe polyarthritis, elevated serum levels of pro-inflammatory factors such as IL-1β, IFN-α, IL-6 and CXCL-10, were found, suggesting the involvement of these mediators in chronic joints and muscle pain (Hoarau et al., 2010). The presence of high levels of these mediators can serve as a biomarker of poor prognosis (Ng et al., 2009). In patients with chronic arthritis, elevated levels of IL-6, GM-CSF and IL- 17 have been observed (Gasque et al., 2015; Chen et al., 2015a). IL-17 can promote chronic extracellular matrix inflammation and bone destruction through the stimulation of TNF-α, IL-1, IL-6, matrix metalloproteinases, and RANKL (Chen et al., 2015a). An association between IL-6 and MCP-1 with high viral load has also been observed (Chow et al., 2011; Reddy et al., 2014) while elevated serum IL-6 and GM-CSF is associated with chronic polyarthralgia (Chow et al., 2011). IL-6 has an important role in the persistence of arthritis, through the regulation of RANKL and the osteoclastogenesis process. This cytokine, secreted by infected human synovial fibroblasts, promotes the recruitment and differentiation of monocytes into osteoclasts, which in turn, will secrete high levels of IL-6, leading to a positive feedback loop that contributes to the progression of arthralgia (Phuklia et al., 2013). In summary, understanding the regulation of inflammatory mediators during alphavirus infection is essential for delineating the delicate balance between both protective immunity and excessive inflammation, which can contribute to immunopathology and severe disease outcomes.

### Innate cellular responses

The innate immune response to alphaviruses is a complex process involving various cells, including macrophages (Suhrbier and La Linn, 2003), dendritic cells (DCs), and natural killer (NK) cells (Lee et al., 2007). These cells are quickly drawn to the infection site, playing a vital role in either protecting the body or contributing to disease development. This carefully coordinated immune response is essential for detecting and containing alphaviral infections early on. Through processes like phagocytosis (where cells engulf the virus), cytokine release (signaling molecules that coordinate the immune response), and direct killing of infected cells, macrophages, dendritic cells, and NK cells work together to limit and ultimately eliminate the virus.

Upon encountering viral particles or infected cells, macrophages initiate a cascade of immune responses, including phagocytosis of infected cells and the release of inflammatory cytokines (-pro and -anti). Some alphaviruses, including VEEV, CHIKV and MAYV, replicate in macrophages (Sourisseau et al., 2007; Gardner et al., 2008) (Cavalheiro et al., 2016). Recent findings have elucidated the diversity of macrophage responses during acute alphavirus infections (Mostafavi et al., 2019). Furthermore, monocytes/macrophages comprise a major component of the cellular infiltrate in alphavirus-infected tissues contributing to the inflammatory process that can last for several years (Jaffar-Bandjee et al., 2009; Her et al., 2010; Labadie et al., 2010). Synovial fluid from individuals with alphavirus-induced polyarthritis presented significantly higher levels of TNFα, IFNy, and macrophage chemoattractant protein (MCP)-1, and such macrophage-derived factors also contribute to the development of arthritis in animal models (Lidbury et al., 2008).

Dendritic cells, known for their antigen-presenting capabilities, contribute significantly to the early detection of viruses. DCs recognize viral antigens and migrate to lymph nodes, where they activate adaptive immune responses by presenting viral antigens to T cells. Additionally, DCs release cytokines that influence the immune milieu, shaping the overall host response to alphaviruses. Some alphaviruses, including sindbis virus (SINV) and VEEV, replicate in myeloid/conventional DCs (cDCs), which can transport viruses to the draining lymph nodes, thereby facilitating viral spread/dissemination (Gardner et al., 2000, Gardner et al, 2008). There is no evidence that CHIKV infects DCs (Sourisseau et al., 2007). Furthermore, CHIKV infection in the absence of DC immunoreceptor (DCIR) results in more severe disease in mice (Long et al., 2013).

Plasmacytoid DCs (pDCs) characterized by their ability to produce high levels of IFNs, emerge as sentinels in the defense against alphaviruses. pDCs recognize viral PAMPs through various PRRs predominantly via endosomal TLRs (Swiecki and Colonna, 2015). The primary effector function of pDCs during alphavirus infection lies in their robust production of IFNs that act in both autocrine and paracrine manners to establish an antiviral state within infected and neighboring cells. Type I IFN can limit CHIKV or MAYV infection of monocytes (Webster et al., 2018; Winkler et al., 2020).

Natural killer cells contribute to the rapid control of viral replication during the early stages of infection, acting as a first line of defense against alphaviruses. Upon encountering alphavirus-infected cells, NK cells undergo activation through a delicate balance of activating and inhibitory signals. The recognition of viral components by activating receptors triggers NK cell cytotoxicity and the release of cytokines. NK cells are capable of directly recognizing and eliminating virus-infected cells through mechanisms such as cytotoxicity (destruction of virus-infected target cells) and the release of cytokines (Brandstadter and Yang, 2011; Maucourant et al., 2019). However, NK cells could have a prominent role in pathological processes by infiltrating synovial tissues, sustaining an inflammatory environment that might contribute to the progression of chronic joint inflammation. After the onset of acute CHIKV infection, NK cells undergo a transient clonal expansion that are correlated with viral load (Petitdemange et al., 2011). In a mouse model of CHIKV, the depletion of NK cells significantly reduced joint pathology corroborating the deleterious role of NK cells in driving virus-induced pathology (Teo et al., 2015).

Understanding the nuanced interactions between these immune cells and the virus is crucial for developing strategies to enhance the innate immune response and help the development of effective antiviral interventions.

## Adaptative immune responses

The adaptive immune response plays a pivotal role in viral clearance and long-term immunity. Upon encountering alphavirus antigens, antigen-presenting cells (APCs) such as DCs and macrophages capture and process viral particles. These APCs migrate to secondary lymphoid organs, where they present viral antigens to naive T lymphocytes via major histocompatibility complex (MHC) molecules. This interaction leads to the activation and differentiation of T cells into effector cells, including cytotoxic CD8^+^ T lymphocytes (CTLs) and helper CD4^+^ T cells (Th cells) that migrate to sites of infection to control virus replication (Wauquier et al., 2011; Kafai et al., 2022).

### Cellular immune responses

Upon recognition of viral peptides presented by MHC class I molecules, CTLs become activated and release cytotoxic granules containing perforin and granzymes, inducing apoptosis in virus-infected cells. This direct cytotoxicity is essential for controlling viral replication and limiting viral spread (Dias et al., 2018; Davenport et al., 2020). Activated CD8^+^ T cell numbers increase in the circulation and in tissues after alphavirus infection (Hoarau et al., 2013; Miner et al., 2015). Most pathogen-specific CTL responses start 3–4 days post-infection, peak by 7–10 days, and then decline (Linn et al., 1998; Wauquier et al., 2011).

Helper CD4^+^ T cells orchestrate the antiviral immune response by providing essential signals for optimal B and CD8^+^ T cell responses. In acute CHIKV infection, patients exhibit transient lymphopenia, a phenomenon that may be partially explained by CD4^+^ T cell apoptosis (Staikowsky et al., 2009). CD4^+^ and CD8^+^ T cells have been shown to infiltrate inflamed joints of mouse models of CHIKV infection (Morrison et al., 2011). Further animal studies have suggested that CD4^+^, but not CD8^+^ T cells, play a major role in mediating the severity of joint inflammation (Teo et al., 2013). In animal models, different T-cell-targeting approaches have been shown to ameliorate CHIKV arthritis severity (Miner et al., 2017; Teo et al., 2017).

### Humoral immune responses

Alphavirus antigens trigger the activation and differentiation of B cells into antibody producing plasma cells. Virus-specific antibodies target viral particles, neutralize their infectivity, promote opsonization and phagocytosis, and activate complement-mediated lysis of infected cells. Understanding the dynamics of antibody responses (the major correlate of immunity), including the kinetics of immunoglobulin class-switching and affinity maturation, provides insights into the potential development of long-lasting immunity.

The current knowledge about the role of antibody-mediated immunity in alphavirus infections has been gained from experimental infection models and cohort studies focused on CHIKV (Torres-Ruesta et al., 2021). Mice lacking B cells (MT, Rag1, Rag2/IL2rg mice) and infected with CHIKV displayed higher and persistent viremia while wild type (WT) mice were able to control the virus (Gardner et al., 2010; Hawman et al., 2013; Poo et al., 2014b).

Generally, IgM antibodies appear five to seven days after symptom onset and are detectable for several weeks post-infection (Kam et al., 2012b; Pierro et al., 2015). Individuals with CHIKV-induced polyarthralgia may have prolonged presence of specific IgM antibodies, likely due to viral persistence (Malvy et al., 2009). Specific IgG antibodies typically appear shortly after IgM detection (4-10 days post-onset) and can persist for several years (Kam et al., 2012c; Nitatpattana et al., 2014; Bozza et al., 2019). IgG3 is the predominant IgG subtype generated upon infection, correlating with effective viral clearance and protection against chronic CHIKV symptoms (Kam et al., 2012c). Several studies have identified structural viral regions recognized by neutralizing antibodies produced during experimental infection (for a comprehensive review, see Torres-Ruesta et al., 2021). The concept of using humoral immunity as a therapeutic approach against alphavirus infection emerged after the isolation of EEEV, WEEV, and VEEV (Torres-Ruesta et al., 2021). Passive transfer of immune serum or monoclonal antibodies has been shown to induce protection against alphavirus infection and disease (Pal et al., 2013; Fox et al., 2015; Jin et al., 2015; Earnest et al., 2019; Powell et al., 2020; Williamson et al., 2020).

## Pathophysiology of alphavirus

The outcome of viral infections results from complex interactions between the pathogen and the host’s immune system. Disease severity and duration often depend on the initial immune response. Therefore, understanding the pathogenesis of acute alphavirus infection is important for developing effective strategies to mitigate morbidity. Alphavirus infections can lead to diverse pathological outcomes, varying between viruses and even among strains of the same virus (Guerrero-Arguero et al., 2021). For example, New World alphaviruses (EEEV, WEEV, VEEV) are typically associated with encephalitis (Strauss and Strauss, 1994), while arthritogenic Old World alphaviruses, including CHIKV, MAYV, RRV, and ONNV, are primarily linked with rheumatic diseases, being a leading cause of infectious arthropathies globally (Liu et al., 2022a). Moreover, viral RNA can persist beyond the acute phase, leading to long-lasting neurological complications or persistent joint pain and inflammation (Atkins, 2013; Zaid et al., 2021). Besides infecting immune cells, these alphaviruses can also infect endothelial cells (Labadie et al., 2010), muscle cells (Morrison et al., 2006; Ozden et al., 2007), periosteum (Heise et al., 2000), and potentially keratinocytes (Pakran et al., 2011). The large repertoire of target cells, combined with the resulting inflammatory immune responses, likely contributes to the acute symptoms induced by these viruses (Schwartz and Albert, 2010).

Over time, viruses and their hosts have co-evolved, resulting in specialized immune recognition mechanisms for detecting viral infection and unique strategies for limiting viral replication (Petitdemange et al., 2015). Alphavirus infections are associated with autoimmune or inflammatory syndromes (Baxter and Heise, 2020) because they trigger a cascade of the host’s innate and adaptive immune responses (Danillo Lucas Alves and Benedito Antonio Lopes da, 2018). Immune factors released from leukocytes (including reactive oxygen, nitrogen species, and prostaglandins), which are part of the host’s defense system, may contribute to the development of viral arthropathies in inflamed tissues (Steer and Corbett, 2003).

Disruptions in the host’s protective mechanisms can also contribute to pathology. Impaired chemotaxis of immune cells can lead to increased viral spread and higher viral loads, triggering exaggerated inflammatory responses (Ong et al., 2014). Conversely, an overly robust chemotactic and inflammatory response, while intended to protect the host, can be detrimental, especially in neurotropic viruses, where such responses may promote neuroinvasion (Ong et al., 2014).

Animal models, especially mice and non-human primates, have proven invaluable for studying alphavirus infections (Gleiser et al., 1962; Skidmore and Bradfute, 2023) and have provided crucial insights into the pathogenesis and immune response (Ryman and Klimstra, 2008). For many years, the infection of mice by SINV or Semliki Forest (SFV) has served as valuable animal model for the study of acute viral encephalitis (Griffin, 1989; Griffin, 2005). Studies on specific alphaviruses like VEEV, EEEV and WEEV in animal models have elucidated various infections pathways and associated pathologies, shedding light on the complexity of alphavirus infections and the host immune response (Baxter and Heise, 2020). Mice infected with virulent strains of VEEV through different routes exhibit distinct invasion patterns and pathological outcomes. Subcutaneous or footpad inoculation of VEEV mirrors natural infection by mosquitoes, with the virus first targeting dermal dendritic cells before invading the central nervous system via axonal transport along olfactory neurons (reviewed by Baxter and Heise, 2020). This process triggers an increase in proinflammatory genes and TLR signaling, compromising the blood-brain barrier integrity and leading to brain infiltration by immune cells (Sharma et al., 2008; Sharma and Maheshwari, 2009; Gupta et al., 2017). In contrast, aerosol or intranasal infection targets the olfactory neuroepithelium, leading to rapid brain infection within 16 hours post-infection (Vogel et al., 1996; Steele et al., 1998; Cain et al., 2017; Bocan et al., 2019). EEEV and WEEV show distinct infection patterns and pathologies, with WEEV entering the brain through the olfactory tract and EEEV occurring hematogenously (Phillips et al., 2013, Phillips et al, 2016; Phelps et al., 2017; Bantle et al., 2019). More virulent WEEV strains cause severe meningoencephalitis characterized by neuronal degeneration, necrosis, edema, and mild mononuclear cell infiltration (Aguilar, 1970; Logue et al., 2009; Phelps et al., 2017), while less virulent strains result in perivascular inflammation but minimal neuronal damage (Logue et al., 2010). Neonatal mice infected with WEEV face high mortality due to severe inflammation and/or necrosis in peripheral tissues (Aguilar, 1970).

Replication in central nervous system (CNS) tissues typically occurs late in the disease progression unless the virus is directly inoculated intracerebrally, which does not accurately mirror the natural course of arthritogenic alphavirus infections (Ryman and Klimstra, 2008). Recent studies have focused on early infection events through subcutaneous virus inoculation to replicate natural transmission via mosquito bites, providing valuable insights into the initial stages of infection and transmission dynamics (Trgovcich et al., 1996). Age-related variations in mouse susceptibility to arthritogenic alphaviruses have emerged as a significant factor, prompting researchers to investigate age-related immune responses in disease models. For instance, while adult mice may exhibit avirulence to arthritogenic alphaviruses like RRV and CHIKV, these viruses can elicit severe illness in humans, indicating the complexity of immune responses across different age groups (Lidbury et al., 2000; Couderc et al., 2008).

Previous studies have shown that CHIKV infection is lethal in neonatal mice due to their immature immune systems (Ziegler et al., 2008). Couderc et al. investigated the susceptibility of adult mice lacking the interferon receptor α/β (IFN-α/βR2/2) to CHIKV isolates from patients on La Reunion Island. They found that these mice developed severe disease characterized by muscle tone loss, lethargy, and mortality within three days of infection (Couderc et al., 2008). This suggests that a robust interferon response in adult mice is protective against fatal CHIKV infection, as observed with other alphaviruses (Grieder and Vogel, 1999; Ryman et al., 2000). Monocytes and CCR2^+^ macrophages seem to play a dual role in CHIKV disease pathophysiology. Treatment with bindarit, an inhibitor of monocyte chemotactic proteins (CCL2, CCL8), attenuated osteoclastogenesis and prevented severe bone loss (Chen et al., 2015b). However, depleting CCR2 macrophages in CHIKV-infected mice increased neutrophil infiltration in the joints, leading to erosive cartilage damage (Poo et al., 2014a; Chen et al., 2015b; Burt et al., 2017).

Various mouse models have been used to study CHIKV pathogenesis and immunity, but they often do not fully recapitulate human disease. Humanized mice, containing human immune system components, have provided valuable insights into human-specific immune responses to arthritogenic alphaviruses like CHIKV. Studies using humanized NSG mice (hu-NSG) infected with CHIKV via mosquito bites showed human-like clinical signs, viremia, immune responses (evidenced by human-specific IgM markers), and histological lesions, highlighting the involvement of the human immune system in the observed pathologies (Hibl et al., 2021). Non-human primates (NHPs), particularly Cynomolgus macaques (CMs), have also been instrumental in studying CHIKV infection due to their similarities with humans in immunology and physiology (Broeckel et al., 2015). Cirimotich et al. (2017) demonstrated that CMs exposed to CHIKV aerosols could develop mild or asymptomatic infections despite detectable virus levels (Cirimotich et al., 2017), mirroring observations in humans (Shah and Baron, 1965; Weaver et al., 2012). By infecting monkeys with different CHIKV strains intradermally, the same group observed varying responses reflecting the virus’s virulence. Peak viremia occurred on the second day post-infection with changes in blood cell counts and enzyme levels (Labadie et al., 2010; Cirimotich et al., 2017), resembling early signs of human infection (Simon et al., 2007). Variations in disease severity were noted between LR2006-OPY1 and Ross strains, highlighting potential strain-specific effects on disease severity in CMs. Furthermore, comparisons between intravenous (Labadie et al., 2010) and intradermal infections yielded similar outcomes, with detectable virus levels for at least six days. Mosquito-borne infection in Indian bonnet macaques (*Macaca radiata*) showed delayed virus levels compared to intravenous infection (Paul and Singh, 1968), indicating the transmission’s mode impact on disease progression. Another study by Broeckel et al., 2015 revealed differences in monocyte and dendritic cell numbers in adult and aged rhesus macaques infected with CHIKV strains, indicating age-related and strain-specific immune responses in NHP models. The same group also demonstrated that macrophages trafficked to lymphoid tissues and harbored CHIKV antigen in the spleen and lymph nodes of infected Cynomolgus macaques (Broeckel et al., 2015).

The immunopathological mechanisms underlying ONNV-induced disease, which shares genetic similarity with CHIKV and induces similar disease manifestations, remain poorly understood (Rezza et al., 2017). Seymour et al. (2013) adapted a CHIKV footpad inoculation and swelling model to investigate ONNV infection (Seymour et al., 2013). They found that wild-type C57BL/6J and S129 mice were resistant, whereas A129 mice (lacking the type I interferon receptor) were susceptible in a dose-dependent manner. The same study also demonstrated that the innate immune response sufficiently controlled low-dose infection (10^3^ pfu), mimicking natural mosquito transmission. Furthermore, while IFN-γ depletion did not increase susceptibility, IFN α/β was essential during the acute disease phase (Seymour et al., 2013). Although much remains unknown about ONNV-induced disease, a study by Partidos et al. (2012) showed that a highly attenuated, recombinant CHIKV vaccine conferred cross-protection against ONNV infection in susceptible A129 mice (Partidos et al., 2012).

Animal models of RRV and MAYV infection have demonstrated that a heightened inflammatory response in the host contributes to the development of myositis and arthritis (Lidbury et al., 2000; de Castro-Jorge et al., 2019). Currently, immunocompetent mouse strains are used to investigate the mechanisms by which RRV induces inflammation (Morrison et al., 2006). Various experimental immunomodulatory approaches have identified key immune cell subsets driving the disease (Mostafavi et al., 2019). Similar to CHIKV infection, monocytes and macrophages play a crucial role in RRV infection. Depletion of these macrophages using liposomal clodronate reduces disease symptoms (Gardner et al., 2010). Furthermore, in C57BL/6 mice infected with RRV, the absence of Rag1^−/−^ B and T cells or B μMT (Ighm^−/−^) B cells does not affect tissue damage compared to wild-type mice, highlighting the importance of the innate immune response in the disease’s immunopathology (Morrison et al., 2006). Subcutaneous administration of the prototypic T48 strain of RRV to 22-day-old C57BL/6J mice induces viremia, peaking at 48–72 hours post-infection. Disease onset typically occurs at 6–7 days post-infection, characterized by severe hind limb function loss, weight loss, lethargy, or partial paralysis. However, mice typically recover completely by 18–20 days post-infection (Morrison et al., 2006). Both musculoskeletal tissues and sera of RRV-infected mice and humans show elevated IL-17 expression. Inhibiting IL-17 has been proposed as a potential strategy to mitigate arthritic alphavirus diseases by reducing proinflammatory gene transcription, cellular infiltration in synovial tissues, and cartilage damage (Mostafavi et al., 2022). Studies have also shown that mice deficient in mannose-binding lectin (MBL), C3, or CR3 complement receptor exhibit less severe rheumatic symptoms than their wild-type counterparts (Morrison et al., 2007, Morrison et al, 2008; Gunn et al., 2012). Furthermore, RRV lacking both E2 glycans results in reduced MBL binding and complement activation, leading to less severe tissue damage compared to wild-type virus infections (Gunn et al., 2018). Powell et al. (2020) demonstrated that E2 glycoprotein-specific human monoclonal antibodies isolated from RRV-infected individuals confer robust immunity in mice, preventing disease. These findings underscore the importance of MBL and viral N-linked glycans in the development of alphaviral disease.

Santos et al. (2019) were the first to establish a comprehensive animal model of MAYV-induced arthritis and myositis using 15-day-old immunocompetent BALB/c mice instead of C57BL/6J mice (Santos et al., 2019). This study also introduced novel methods to assess pathology, including measurements of grip strength, endurance, and mechanical hypernociception. During MAYV infection, inflammation is characterized by a significant increase in pro-inflammatory cytokines through NLRP3 inflammasome activation, elevated CCL2 levels, and substantial recruitment of CCR2^+^ macrophages to the infection site (Santiago et al., 2015; Tappe et al., 2016; Santos et al., 2019, Santos et al., 2024). Additionally, de Castro-Jorge et al. (2019) demonstrated that MAYV can replicate in bone marrow-derived macrophages and trigger inflammasome activation in a C57BL/6 mouse model. Despite the use of various animal models for MAYV infection, the underlying mechanisms of the disease’s pathogenesis remain largely unknown (de Castro-Jorge et al., 2019).

In humans, the transmission of alphaviruses occurs through the bite of infected mosquitoes, such as *Haemagogu*s for MAYV, *Aedes* spp. for CHIKV, and *Culex* spp. or *Aedes* spp. for RRV or viral encephalitis. Once transmitted, alphaviruses like MAYV, CHIKV, RRV, and others replicate initially in the skin’s fibroblasts before disseminating to other organs like the spleen, lymph nodes, and microvasculature. This dissemination can affect organs such as the liver, muscles, and joints, establishing the infectious process within the body (Atkins, 2013). Additionally, certain alphaviruses can enter the central nervous system (CNS), where they replicate in astrocytes, glial cells, and neurons, potentially leading to fatal encephalitis (Gauri et al., 2012; Lindsey et al., 2018).

Encephalitic viruses, such as WEEV, VEEV, and EEEV, replicate within neuronal cells, triggering the production of pro-inflammatory cytokines that weaken the blood-brain barrier. EEEV infection is generally more severe than WEEV and VEEV, with a higher risk of fatality (Baxter and Heise, 2020). EEEV infects neurons through a vascular route (Vogel et al., 2005), leading to severe symptoms such as high fever, headache, vomiting, seizures, and potential long-term neurological complications (Deresiewicz et al., 1997; Powers and Roehrig, 2011). WEEV can cause fever, headache, neck stiffness, photophobia, nausea, vomiting, weakness, tremors, and altered behavior (Liu et al., 2018), with 15-30% of patients experiencing secondary neurological damage (Aguilar et al., 2011). VEEV infection is initially asymptomatic, followed by fever, headache, nausea, vomiting, diarrhea, joint and muscle pain, and back pain. VEEV replicates in both lymphoid and non-lymphoid tissues, causing inflammation characterized by tissue necrosis (Watts et al., 1998). CNS invasion by VEEV results in neuroinflammation, neurodegeneration, gliosis, and neutrophil vacuolization. Aerosol exposure to VEEV can cause upper respiratory symptoms and may lead to encephalitis (Rusnak et al., 2018). Percutaneous transmission of VEEV has also been reported in humans, with hematological analysis revealing lymphocytosis, leukopenia, and neutropenia (Rusnak et al., 2018).

Arthritogenic alphaviruses, including RRV, CHIKV, MAYV, SFV, SINV, ONNV, and Barmah Forest virus, significantly impact the joints and may cause neurological manifestations. However, limited epidemiological data and a lack of comprehensive understanding of their pathophysiology hinder the development of effective prevention and control strategies (Suhrbier et al., 2012). These infections have two distinct phases: acute and chronic. The acute phase typically manifests 3-10 days post-infection, with a viremic period lasting 4-7 days. Clinical manifestations are nonspecific, often leading to confusion with other arboviral infections (Suhrbier et al., 2012; Atkins, 2013). The frequency of clinical disease varies among viruses. Most RRV and SINV infections are asymptomatic, while up to 80% of CHIKV and MAYV infections result in clinical symptoms (Harley et al., 2001; Brummer-Korvenkontio et al., 2002; Azevedo et al., 2009; Vijayakumar et al., 2011; Suhrbier et al., 2012; Endy, 2020).

The cause of alphavirus-induced chronicity remains unclear. It is hypothesized that the persistence of viral antigen or residual proinflammatory mediators in previously infected tissues may contribute to chronic inflammation (Assunção-Miranda et al., 2013). Most acute symptoms typically resolve within one to two weeks. However, arthralgia and arthritis may persist for months to years, causing significant physical, emotional, mental, and financial burdens (Baxter and Heise, 2020). Symptom persistence correlates with the severity of inflammation in joints and muscles, the extent of tissue damage, and the presence of viral products within tissues (Baxter and Heise, 2018). Chronicity occurs in 25-55% of individuals infected with RRV, SINV, or CHIKV. Barmah Forest virus infections have lower chronicity rates, while data on Mayaro virus chronicity are limited (Suhrbier and La Linn, 2004; Assunção-Miranda et al., 2013). Synovial biopsies from patients with chronic CHIKV-induced polyarthritis showed synovitis with inflammation composed of macrophages, NK cells, CD4^+^ T cells, and plasma cells, along with synovial lining hyperplasia and vascular proliferation (Hoarau et al., 2010; Ganu and Ganu, 2011). Synovial effusions in chronic CHIKV arthritis mainly consisted of CD14^+^ monocytes/macrophages, fewer activated NK cells and CD4^+^ T cells, with less than 5% testing positive for CHIKV (Hoarau et al., 2010). Synovial effusions from chronic RRV disease cases also contained mononuclear cells, predominantly CD4^+^ T cells (Fraser and Becker, 1984).

Infection with Old World alphaviruses can lead to rare but possible fatalities, particularly in neonatal or elderly individuals with comorbidities (Economopoulou et al., 2009; Tandale et al., 2009). Fatal cases are often associated with respiratory, renal, hepatic, and neurological complications, especially in individuals with pre-existing conditions such as hypertension, respiratory, or cardiovascular disease (Economopoulou et al., 2009; Chua et al., 2010; Hoz et al., 2015; Mercado et al., 2018). Postmortem examinations of CHIKV infections have revealed diverse pathological findings, including acute tubulointerstitial nephritis, viral pneumonia, hepatocellular necrosis, and acute pericarditis (Mercado et al., 2016, Mercado et al, 2018; Reilly et al., 2020). Neurological complications are the most fatal among atypical manifestations of CHIKV infection (Economopoulou et al., 2009; Tandale et al., 2009). Patients with CHIKV-induced neurological complications exhibit elevated levels of various inflammatory markers in cerebrospinal fluid compared to those with non-CHIKV-induced neurological disease (Kashyap et al., 2014). Maternal-fetal transmission of CHIKV carries a pooled risk of 2.8% for neonatal death, with infants infected later in gestation exhibiting various symptoms such as joint edema, petechiae, rash, thrombocytopenia, disseminated intravascular coagulopathy, and encephalopathy, potentially leading to long-term disabilities (Touret et al., 2006; Gerardin et al., 2008).

## Immune evasion

Alphavirus immune evasion strategies play a crucial role in their pathogenesis and disease development. Key viral RNA structures significantly influence translation, immune evasion, and regulation of viral synthesis within the host’s innate immune system (Kutchko et al., 2018). While both innate and adaptive immune responses are essential for clearing viral infections, subtle variations in the host’s immune response can affect disease severity (Chan and Ng, 2017). Alphaviruses have evolved diverse mechanisms to suppress host responses during cellular replication, relying on early evasion tactics to enhance permissibility in the infected host (Landers et al., 2021).

**Figure 2:**
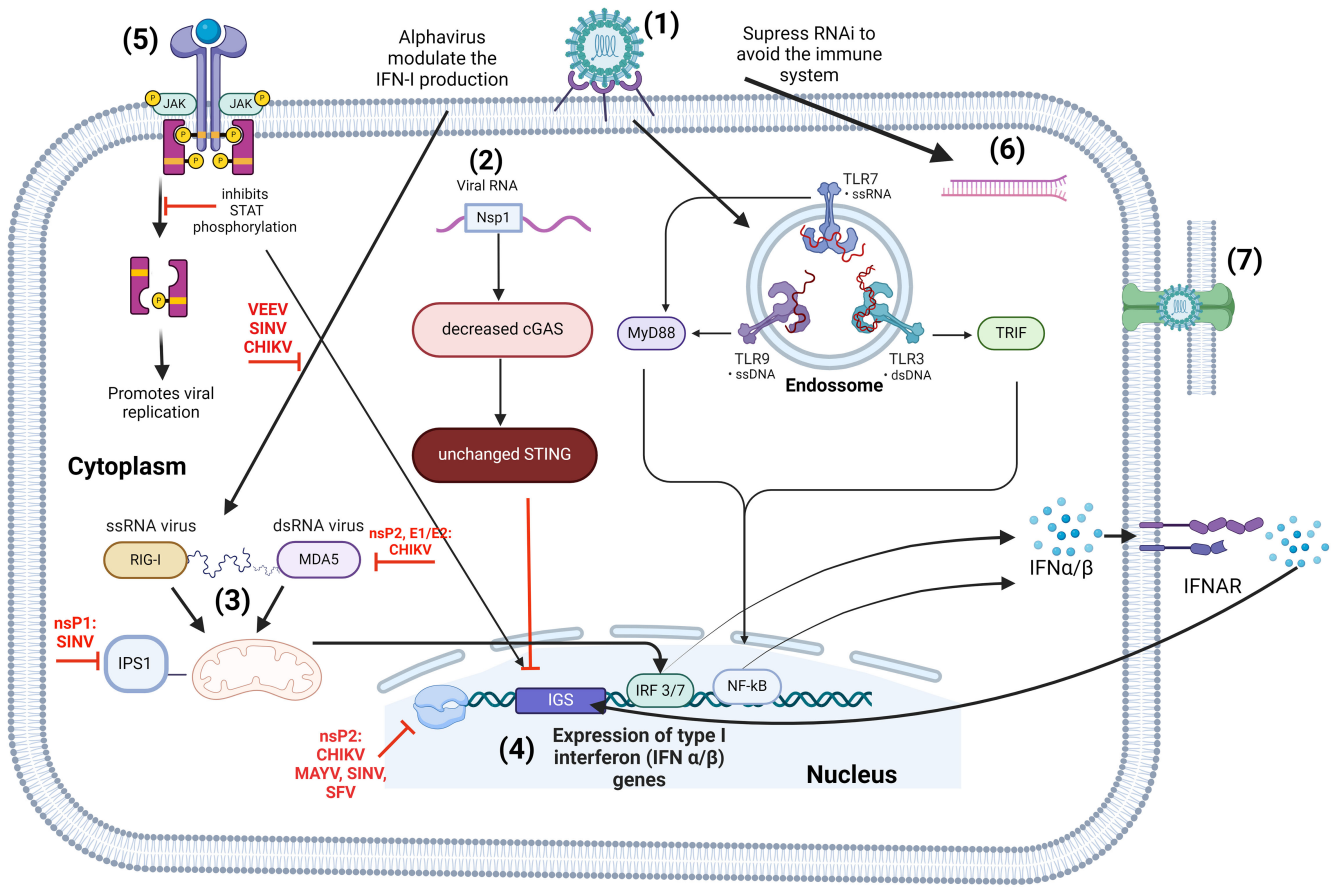
Alphavirus immune evassion with emphasis on type I IFN responses. (1) Alphavirus attachment and entry are mediated by host pattern recognition receptors (PRRs), such as RIG, NOD, and TLRs, triggering the production of IFN-I. Once inside the host’s cells, alphaviruses interfere with the IFN-I system at various levels: (2) Inhibition of the cGAS-STING pathway. Following CHIKV infection, there is a significant decrease in cGAS expression, while STING expression remains relatively stable. The non-structural protein nsPl interacts with STING, affecting the activation of IFNs and the activation of the IFN-β promoter, typically controlled by cGAS-STING pathway; (3) CHIKV nsP2 and E1/E2, and SINV nsPI, inhibit the activation of the IFNβ-promoter triggered by the MDA5/RIG-I receptor signaling pathway; (4) The nsP2 from CHIKV, MAYV, SINV, SFV influence ISGs through modifications such as palmitoylation and phosphorylation to hinder interferon α/β and ISG production; (5) Inhibition of JAK-STAT signaling: CHIKV disrupts the phosphorylation of STAT1, essential for IFN-induced JAK-STAT signalling, aiding in immune evasion and enhancing viral replication. (6) Alphaviruses develop viral RNAi suppressors to evade RNAi-based defense mechanisms in eukaryote, as exemplified by the Semliki virus. (7) Alphaviruses like CHIKV employ strategies to avoid the adaptive immune response, including the formation of stable cellular extensions that shield the virus from neutralizing antibodies and facilitate efficient intercellular transmission. [Created with BioRender.com with license no. BG270SYVXK.].

The innate immune system is the primary defense against viral infections, but pathogens have evolved strategies to counteract this response (Webb et al., 2020; Elrefaey et al., 2021). Interferon type I is a major target for viral evasion due to its critical role in activating both innate and adaptive immune responses, essential for controlling viral infections (Liu et al., 2022b). Viruses target both upstream and downstream components of the interferon signaling pathways, as summarized in **Figure 2**. The cGAS-STING signaling pathway is essential for triggering interferon (IFN) gene expression during tissue damage, cellular stress, and infections (Decout et al., 2021). CHIKV infection significantly decreases cGAS expression, while STING expression remains unchanged (Webb et al., 2020; Decout et al., 2021). Interactions between the viral non-structural protein Nsp1 and STING influence IFNs induction and IFNβ promoter activation, normally induced by the cGAS-STING pathway (Webb et al., 2020; Decout et al., 2021). Pattern recognition receptors like RIG, NOD, and TLRs initiate signaling cascades leading to IFN-I production (Bae et al., 2019). ISGs are crucial for controlling CHIKV, RRV, SINV, and ONNV replication (Ryman et al., 2000; Lenschow et al., 2007; Bréhin et al., 2009; Seymour et al., 2013). Mice lacking IFNs are more susceptible to CHIKV infection, leading to widespread viral dissemination, including the central nervous system (Couderc et al., 2008). Viperin, an ISG product, is necessary for the antiviral response to CHIKV, with its expression correlating with viral load in infected patients. CHIKV infection also activates IRF3, promoting IFN-β and ISG transcription (White et al., 2011). In SINV infection, IFNs expression depends on IRF3 activation via MDA5 (Burke et al., 2009), while RRV is recognized by PRRs (Neighbours et al., 2012). TLR7-deficient mice infected with RRV exhibit increased tissue damage and viral titers, along with elevated antibody responses with reduced neutralizing activity and epitope affinity compared to wild-type mice (Neighbours et al., 2012).

Viral modifications, such as phosphorylation and palmitoylation, alter protein hydrophobicity, often anchoring them to the cell membrane (Liu et al., 2022b). In an ONNV mouse model, deficiency in STAT, which mediates IFN signaling, exacerbates disease severity (Seymour et al., 2013). EEEV, SINV, and CHIKV inhibit STAT phosphorylation and translocation to the nucleus, preventing the upregulation of IFN-α/β and the synthesis of ISGs (Simmons et al., 2009; Yin et al., 2009; Fros et al., 2010). CHIKV infection hampers STAT1 phosphorylation, a crucial component of JAK-STAT immune signaling, leading to dysregulation in inflammatory mediators and potentially contributing to autoimmune diseases and cancer pathways (Hu et al., 2021). Studies suggest that CHIKV’s non-structural protein 2 (nsP2) modulates IFN-induced JAK-STAT signaling (Stark and Darnell, 2012; Göertz et al., 2018). Additionally, CHIKV evades CD8^+^ T lymphocyte antiviral responses and establishes chronic infections in joint-associated tissues. This is achieved through CHIKV-infected cells producing nsP2, which inhibits MHC-I presentation on antigen-presenting cells, preventing CD8^+^ T cell recognition (Poo et al., 2014b; Davenport et al., 2020). CHIKV infection also results in persistent high levels of viral replication in elderly individuals, primarily due to immunological senescence, which affects antiviral immunity, particularly in CD4^+^ T lymphocytes, impairing the host’s response (Ware et al., 2023).

Following alphavirus infection, viral genetic material replication induces severe cytopathic changes in host cells. Viral infections disrupt protein folding in the endoplasmic reticulum, suggesting that this virus family can suppress the host’s translational shutdown pathway, selectively inhibiting both host and viral messenger RNA synthesis (Garmashova et al., 2007). Consequently, the virus orchestrates activities that antagonize protein synthesis, efficiently translating viral messenger RNA while shutting down host cell translation (Rathore et al., 2013). RNA interference (RNAi) is a conserved mechanism in eukaryotes for post-translational gene silencing through double-stranded small RNAs (de França et al., 2010). This serves as a defense against viral infections, although viruses can counteract this defense by producing viral RNAi suppressors (Li and Ding, 2022; Liu et al., 2022b). Qian et al. (2020) demonstrated that the capsid protein of the Semliki virus can counteract RNAi in mammalian cells (Qian et al., 2020).

Old World alphaviruses have a significant impact on vertebrate cell cultures, causing extensive cell death (cytopathic effects) (Fros and Pijlman, 2016). The nonstructural protein nsP2 from SINV and SFV plays a key role in this process. NsP2 is not only involved in viral RNA replication and transcription, but it also directly inhibits the host cell’s ability to produce messenger RNA (mRNA) by promoting the degradation of RPB1, a critical subunit of the RNA polymerase complex (Garmashova et al., 2006; Akhrymuk et al., 2018). Interestingly, mutations in nsP2 and another nonstructural protein, nsP3, can affect these interactions. Some mutations enhance the host’s IFNs response without impacting virus replication (Akhrymuk et al., 2018). MAYV’s nsP2 protein also interacts with RPB1 and another protein involved in transcription initiation (TFIIS), further influencing host cell transcription and immune responses (Ishida et al., 2021). Mutations in conserved regions of alphaviruses like CHIKV and SFV can weaken viral RNA replication, leading to reduced cell death (cytopathic activity) (Rikkonen et al., 1992; Fros et al., 2013). The nsP2 protein can enter the host cell’s nucleus through a specific signal sequence (NLS). Mutations affecting this NLS significantly inhibit nuclear translocation but do not necessarily reduce the severity of cell death or cell shutdown (Fazakerley et al., 2002; Pal et al., 2014). While this mutation does not weaken the virus in hamster cells, it does decrease nerve damage (viral neuropathy) in mice (Kam et al., 2012a; Pal et al., 2014). New World alphaviruses like VEEV and EEEV exhibit different cytotoxic effects compared to Old World alphaviruses. VEEV and EEEV replicons induce fewer cell death changes but establish persistent viral RNA replication (Petrakova et al., 2005). In these viruses, host transcription shutdown relies on the presence of the viral capsid protein, which accumulates in the cytoplasm of infected cells and interferes with the antiviral response (Garmashova et al., 2007). The capsid protein inhibits the cell’s production of messenger RNA (mRNA) and ribosomal RNA, thereby downregulating RNA synthesis (Garmashova et al., 2007). WEEV also inhibits host transcription through both nsP2 and capsid proteins, supporting the theory that WEEV evolved from ancestors like SINV and EEEV (Garmashova et al., 2007).

Findings on alphavirus evasion of the adaptive immune system is limited, but existing studies provide valuable insights for future investigations. CHIKV has been shown to induce the formation of stable cell-to-cell connection tunnels, protecting it from neutralizing antibodies and facilitating efficient intercellular transmission *in vitro* and *in vivo* (Yin et al., 2023). Using a co-culture system, Yin et al. (2023) observed differences in CHIKV transmission between cell-to-cell and free infection. Knocking out the MXRA8 receptor in mouse embryonic fibroblasts (MEF) significantly reduced free CHIKV infection but did not affect intercellular tunnel formation or cell-to-cell transmission. This suggests that the high concentration of virus particles at tunnel contact sites may bypass the need for MXRA8 in target cell infection. However, the endocytic pathway was crucial for intercellular transmission, as inhibiting key components like dynamin and Rab5DN reduced CHIKV cell-to-cell transmission. Interestingly, while tetherin typically inhibits virus release into the extracellular environment, it was found to hinder CHIKV cell-to-cell transmission by reducing the virus’s presence at tunnel contact sites (Yin et al., 2023). CHIKV also undergoes mutations that allow it to evade antibody responses and neutralization (Kutchko et al., 2018). These mutations lead to antibody evasion and hinder viral clearance. Pal et al. (2014a) studied CHIKV strains that escaped monoclonal antibody therapy, investigating their clinical traits, stability, and prevalence in arthropod hosts (Pal et al., 2014b). Their findings further support the concept of antibody response evasion (Yin et al., 2023).

## Nervous system complications

**Figure 3:**
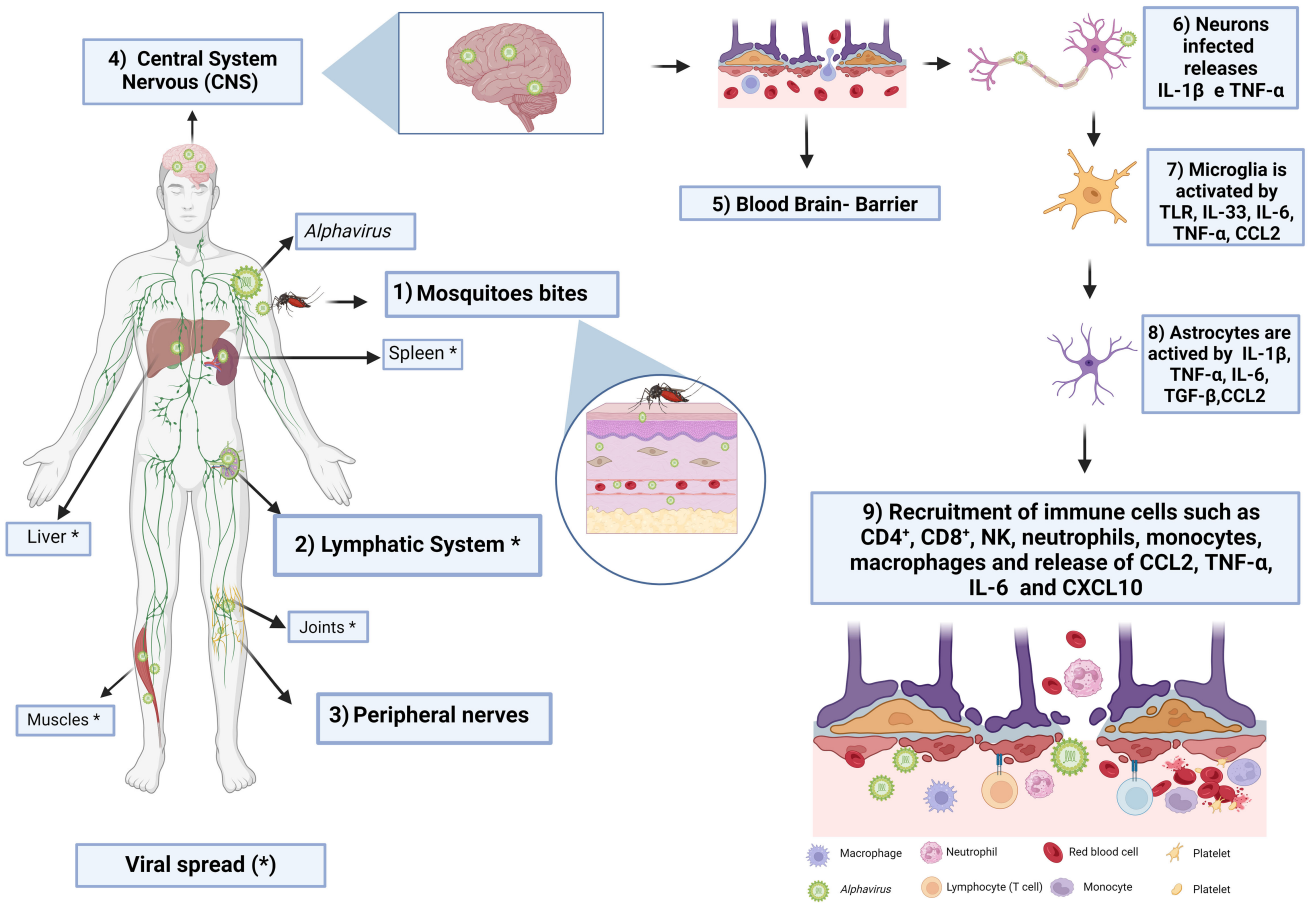
Proposed mechanism of arthritogenic viruses’ pathogensis: from skin entry to neuroinflammation. (1) When a virus-infected mosquito bites a human, it injects saliva containing the virus through its proboscis, breaking the skin barrier. The virus initially infects dendritic cells and fibroblasts, triggering the activation of the innate- immune system through pattern recognition receptors (PRRs) like Toll-like receptors (TLRs) and RIG-I type receptors (RLRs). This initiates a series of immune responses, including the recruitment of macrophages and other immune cells, as well as the production of pro-inflammatory cytokines (such as IL-6 and TNF-α), chemokines (CCL2, CXCL10), and interferons (IFN-α. and IFNβ); (2) despite the body’s efforts to contain the virus, it reaches the nearest lymph node with the help of migratory immune cells like dendritic cells. In the lymph nodes, the virus replicates intensely before .entering the bloodstream, causing viremia. From there, the virus spreads to various organs like the liver, muscles, joints, spleen, and, in severe cases, the brain; (3) the virus reaches the brain through peripheral nerves and the bloodstream; (4) once in the Central Nervous System (CNS), (5) the virus compromises the blood-brain barriers integrity, using mechanisms like “Trojan horse,” transcytosis, or direct disruption to enter the sterile environment; (5) infected neurons release signals like TNF-α and IL-1β, activating glial cells; (6) microglia, specialized macrophages acting as sentinel cells, are the first to respond to neuronal infection by recognizing danger signals from neurons through receptors like Toll-like receptors; (8) this triggers the activation of microglia, followed by astrocyte activation. Activated astrocytes release various molecules like IL-1β, TNF-α, IL-6, IFN- γ, TGF-β, and CCL2, which promote neuroroinflammation and modulate blood-brain barrier permeability; (9) astrocytes further stimulate an inflammatory response by recruiting immune cells like CD4+T, CD8+T, NK cells, neutrophils, monocytes, and macrophages to the site of infection, releasing chemokines and cytokines like CXCL10, CCL2, TNF-α, and IL-6. [Created with Biorender.com with license no. DJ272BIEJ1.].

New World alphaviruses like WEEV, VEEV, and EEEV are known to cause neurological disorders (Griffin, 1992; Zacks and Paessler, 2010). Recently, there has been an increase in neurological issues linked to viruses not typically associated with such complications. Notably, CHIKV, an Old-World alphavirus primarily known for causing joint pain, has been implicated in unusual and severe neurological problems like meningoencephalitis and encephalopathy (Economopoulou et al., 2009; Lebrun et al., 2009), as summarized in **Figure 3**. Neurological complications from CHIKV infection have the highest mortality rate among its atypical manifestations (Economopoulou et al., 2009; Tandale et al., 2009). Patients with CHIKV-induced neurological complications show elevated levels of cytokines (TNF-a, IFN-a, IL-6) and chemokines (CCL2, CCL5, CCL7, CXCL9) in their cerebrospinal fluid compared to those without neurological symptoms (Kashyap et al., 2014). Recently, de Souza et al. (2024) identified CCL-2 as crucial for recruiting monocytes into the brain, including the CD14^+^CD16^+^ subset, potentially serving as a “Trojan horse” mechanism for CHIKV entry into the CNS (de Souza et al., 2024). Reports of CHIKV affecting the CNS emerged during epidemics in Thailand and India (Thiruvengadam et al., 1965; Nimmannitya et al., 1969), leading to neuro-chikungunya with symptoms like meningo-encephalopathy, seizures, encephalomyelitis, Guillain-Barré syndrome, and optic neuritis (Mittal et al., 2007; Mehta et al., 2018) across various age groups (Torres et al., 2016; Cerny et al., 2017). A study of 33 CHIKV-exposed infants in La Réunion found that about 50% had neurodevelopmental delays (Gérardin et al., 2014). Cases of encephalopathy, microcephaly, and cerebral palsy were reported, some emerging after birth, similar to ZIKV-infected children. In another group of 87 CHIKV-positive children, 20% showed developmental delays, including cognitive impairments, 3.5 to 4.5 years post-infection (Cle et al., 2020). Two proposed mechanisms for CHIKV-related CNS complications are direct CNS infection through the choroid plexus (Cerny et al., 2017) and harmful inflammatory responses (Das et al., 2015; Inglis et al., 2016). This information was further corroborated by de Souza et al. (2024) that has demonstrated changes in sPECAM-1 and PECAM-1 levels in infected cells suggesting disruptions in endothelial interactions affecting blood-brain barrier permeability. Metabolomic and proteomic profiles showed immune dysregulation, inflammation, and endothelial damage, with alterations in various protein levels. The hyperinflammation observed in fatal cases could lead to endothelial injury, coagulation cascade dysregulation, and reduced vasopressin levels contributing to hemodynamic disturbances (de Souza et al., 2024). In contrast, MAYV infections that are phylogenetically related to CHIKV, rarely cause CNS disorders, with limited knowledge on the molecular pathways of MAYV’s CNS access in humans (Bengue et al., 2021). Similarly, cases of neurological conditions and viral meningoencephalomyelitis have been documented in individuals infected with the Semliki Forest virus (SFV) (Willems et al., 1979).

Animal studies have shown that CHIKV targets the choroid plexus and leptomeninges in the CNS (Couderc et al., 2008), while MAYV can infect the brains of young wild-type and type I interferon receptor-deficient mice (Figueiredo et al., 2019). CHIKV infects a wider range of CNS cell types, including human neural progenitor cells (hNPCs), at a higher rate than MAYV, although both viruses can infect hNPCs (Bengue et al., 2021). This is significant because ZIKV infection of hNPCs has been linked to disruptions in neurogenesis and severe neurological issues in newborns (Liang et al., 2016; Ferraris et al., 2019). Astrocytes, which are essential regulators of brain homeostasis, are infected by neurotropic arboviruses like CHIKV, leading to neuroinflammatory responses and potential neurological impairments (Inglis et al., 2016). Bengue et al., 2021 revealed that MAYV-infected astrocytes exhibit more extensive modulation of immune gene expression than CHIKV-infected cells, with distinct patterns of upregulated PRRs and chemokines (Bengue et al., 2021). The differential response of astrocytes to MAYV and CHIKV, including the impact on interferon production and inflammatory mediators like IL-6, sheds light on the varying neurovirulence mechanisms of these viruses (Priya et al., 2014; Bengue et al., 2019). Pretreating astrocytes with interferons may restrict MAYV replication, suggesting potential therapeutic strategies (Bengue et al., 2021). Interestingly, CHIKV did not affect IRF3 expression, a key factor in IFN induction, while MAYV infection enhanced it. Bengue et al. also demonstrated a pronounced induction of IFIH1 and DDX58 transcripts by MAYV compared to CHIKV, indicating significant upregulation of these PRRs by both viruses. Their study also uncovered the upregulation of CXCL10, CXCL11, and CCL5 during viral infections, suggesting a robust inflammatory response. Chemokines like CXCL10 can have dual roles in virus-induced neuropathy, being both neuroprotective and potentially neuro-pathogenic (Bengue et al., 2021).

Neuroinvasive SINV infection alters sphingolipid (SL) levels, impacting viral replication (Avraham et al., 2023). Treating mice with GZ-161 prevented the SINV-induced rise in serum SL levels, suggesting that elevated SLs may trigger an immune response against SINV. Elevated SL levels are linked to immune responses, particularly IFNs (Albeituni and Stiban, 2019). GZ-161 appears to modulate NKT cell activation, influencing the immune response in SVNI-infected mice. Inhibiting excessive inflammatory responses through iNKT cell modulation may benefit the management of acute viral encephalitis (Avraham et al., 2023). This aligns with previous findings implicating pathogenic Th17 cells and CD4^+^ and CD8^+^ T cells in fatal SINV encephalitis, suggesting GZ-161’s protection may come from suppressing harmful immune responses rather than solely inhibiting viral replication (Rowell and Griffin, 2002; Kulcsar et al., 2014; Kulcsar and Griffin, 2016).

Neurotropic viruses can breach the CNS through multiple pathways, not just hematogenous spread. Phillips et al. (2016) identified that VEEV, WEEV, and potentially EEEV enter the CNS at specific sites lacking the blood-brain barrier (BBB) (Phillips et al., 2016). While early viral signals were not detected in certain CNS tissues or the olfactory bulb, infection of the olfactory bulb occurred later during CNS dissemination via neuronal pathways from circumventricular organs entry points (Ghosh et al., 2011; Phillips et al., 2016). Various neuroinvasion routes have been proposed, including endothelial routes for VEEV (Gorelkin, 1973) and EEEV (Honnold et al., 2015), the olfactory tract (Charles et al., 1995; Vogel et al., 1996), leukocyte infection (CHIKV and EEEV) (Vogel et al., 2005; Couderc et al., 2008), and retrograde axonal transport (SINV) (Cook and Griffin, 2003). WNV can infect the CNS through axonal transport from peripheral neurons or interneuronally via the spinal cord (Samuel et al., 2007). VEEV uses the olfactory pathway, replicating in the nasal mucosa to transiently disrupt the BBB and facilitate CNS invasion (Schäfer et al., 2011). Circumventricular organs, including the anteroventral third ventricle (AV3V), the organum vasculosum lamina terminalis within the AV3V (McKinley et al., 2003), and the hypothalamus, are essential for virus invasion, with the hypothalamus being a common target (Morita and Miyata, 2012; Miyata, 2015). Hypothalamic neurons in the hypophyseal portal system are susceptible to alphavirus invasion, impacting neuroendocrine hormone secretion (Phillips et al., 2016).

The breakdown of the BBB after VEEV inoculation is linked to infection outcomes (Phillips et al., 2016). WEEV infections can cause long-lasting neurological effects like Parkinsonism, even after encephalitis recovery, with some cases reporting severe neurological sequelae (Schultz et al., 1977). Blocking BBB opening can mitigate VEEV’s CNS penetration (Charles et al., 1995). Altering the BBB is pivotal in encephalitis progression. WNV alters BBB permeability through cytokines and matrix metalloproteinases (Wang et al., 2004; Arjona et al., 2007; Suthar et al., 2013), leading to inflammation and CNS entry. This process involves TLR3-mediated TNF-α production, immune cell infiltration, and MIF-induced metalloproteinase activation (Wang et al., 2004; Arjona et al., 2007; Yu et al., 2007; Verma et al., 2010). Some viruses use infected immune cells as a “Trojan Horse” to cross the brain endothelium and induce inflammation (Dropulić and Masters, 1990). This mechanism, along with direct infection of endothelial cells, has been proposed for WNV entry into the CNS (Verma et al., 2009).

All these findings contribute to our understanding of the complex interactions between arboviruses and the central nervous system, offering insights into the mechanisms underlying virus-induced neuropathology and immune responses.

## Impact of coinfection on the nervous system outcome

Co-infection with multiple alphaviruses or an alphavirus and another pathogen can lead to more severe neurological disease. While no studies directly address the impact of coinfection on the nervous system, the available findings allow us to hypothesize potential mechanisms. Alphavirus coinfections may worsen neurological outcomes by increasing inflammation, promoting neurodegeneration, and disrupting normal immune responses in the CNS. The immune response to alphavirus coinfections within the CNS involves the activation of microglia, astrocytes, and infiltrating immune cells, which release a surge of inflammatory molecules (cytokines and chemokines) (Wongchitrat et al., 2024). This creates a highly inflammatory environment that can lead to complex neurological problems. Excessive immune activation triggers widespread inflammation in the brain and spinal cord, damaging neurons and disrupting their function (Baxter and Heise, 2020). Chronic neuroinflammation disrupts the CNS environment, damaging neurons, impairing synaptic transmission, and compromising overall neuron health (Kolliker-Frers et al., 2021). The persistent activation of immune cells and the release of neurotoxic molecules may contribute to neurodegenerative processes, further worsening neurological impairment (Bantle et al., 2019; Adamu et al., 2024). Alphavirus coinfections can also disrupt the BBB, a protective layer that normally shields the CNS. This disruption allows more immune cells and viral particles to enter the CNS, amplifying neuroinflammation and neurodegeneration (Sian-Hulsmann and Riederer, 2024). Additionally, different alphaviruses interacting within a coinfection may increase their neurovirulence, raising the risk of neurological complications. Understanding the dynamics of coinfections is pertinent for improving the diagnosis, treatment, and management of alphavirus-related neurological diseases.

## Conclusion

In conclusion, alphaviruses can in some instances invade the nervous system, causing a range of atypical neurological complications, from mild encephalitis to severe conditions like Guillain-Barré syndrome and chronic impairments. Co-infections with other pathogens may exacerbate these neurological issues by disrupting the blood-brain barrier, dysregulating the immune system, and potentially influencing alphavirus replication. Investigations on risk factors for atypical neurological outcomes, the role of co-infections, and developing improved diagnostic tools for early detection are being actively pursued. The increasing prevalence of alphaviruses due to climate change and global travel underscores the importance of monitoring and understanding these complex diseases to protect vulnerable populations.

## References

[B1] Adamu A. Li S. Gao F. Xue G. (2024). The role of neuroinflammation in neurodegenerative diseases: current understanding and future therapeutic targets. Front. Aging Neurosci. 16. doi: 10.3389/fnagi.2024.1347987 PMC11045904 38681666

[B2] Aguilar M. J. (1970). Pathological changes in brain and other target organs of infant and weanling mice after infection with non-neuroadapted Western equine encephalitis virus. Infect. Immun. 2, 533–542. doi: 10.1128/iai.2.5.533-542.1970 16557874 PMC416047

[B3] Aguilar P. V. Estrada-Franco J. G. Navarro-Lopez R. Ferro C. Haddow A. D. Weaver S. C. (2011). Endemic Venezuelan equine encephalitis in the Americas: hidden under the dengue umbrella. Future Virol. 6, 721–740. doi: 10.2217/fvl.11.5 21765860 PMC3134406

[B4] Akhrymuk I. Frolov I. Frolova E. I. (2018). Sindbis Virus Infection Causes Cell Death by nsP2-Induced Transcriptional Shutoff or by nsP3-Dependent Translational Shutoff. J. Virol. 92 (23), e01388-18. doi: 10.1128/JVI.01388-18 30232189 PMC6232463

[B5] Albeituni S. Stiban J. (2019). Roles of ceramides and other sphingolipids in immune cell function and inflammation. Adv. Exp. Med. Biol. 1161, 169–191. doi: 10.1007/978-3-030-21735-8_15 31562630

[B6] Arjona A. Foellmer H. G. Town T. Leng L. McDonald C. Wang T.. (2007). Abrogation of macrophage migration inhibitory factor decreases West Nile virus lethality by limiting viral neuroinvasion. J. Clin. Invest. 117, 3059–3066. doi: 10.1172/JCI32218 17909632 PMC1994625

[B7] Assunção-Miranda I. Cruz-Oliveira C. Da Poian A. T. (2013). Molecular mechanisms involved in the pathogenesis of alphavirus-induced arthritis. BioMed. Res. Int. 2013, 973516. doi: 10.1155/2013/973516 24069610 PMC3771267

[B8] Atkins G. J. (2013). The pathogenesis of alphaviruses. ISRN Virol. 2013, 861912. doi: 10.5402/2013/861912

[B9] Avraham R. Melamed S. Achdout H. Erez N. Israeli O. Barlev-Gross M.. (2023). Antiviral activity of glucosylceramide synthase inhibitors in alphavirus infection of the central nervous system. Brain Commun. 5 (3), fcad086. doi: 10.1093/braincomms/fcad086 37168733 PMC10165247

[B10] Azevedo R. S. Silva E. V. Carvalho V. L. Rodrigues S. G. Nunes-Neto J. P. Monteiro H.. (2009). Mayaro fever virus, Brazilian Amazon. Emerg. Infect. Dis. 15, 1830–1832. doi: 10.3201/eid1511.090461 19891877 PMC2857233

[B11] Bae S. Lee J. Y. Myoung J. (2019). Chikungunya virus-encoded nsP2, E2 and E1 strongly antagonize the interferon-β Signaling pathway. J. Microbiol. Biotechnol. 29, 1852–1859. doi: 10.4014/jmb.1910.10014 31635445

[B12] Bantle C. M. Phillips A. T. Smeyne R. J. Rocha S. M. Olson K. E. Tjalkens R. B. (2019). Infection with mosquito-borne alphavirus induces selective loss of dopaminergic neurons, neuroinflammation and widespread protein aggregation. NPJ Parkinsons Dis. 5, 20. doi: 10.1038/s41531-019-0090-8 31531390 PMC6744428

[B13] Battisti V. Urban E. Langer T. (2021). Antivirals against the chikungunya virus. Viruses 13 (7), 1307. doi: 10.3390/v13071307 34372513 PMC8310245

[B14] Baxter V. K. Heise M. T. (2018). Genetic control of alphavirus pathogenesis. Mamm Genome 29, 408–424. doi: 10.1007/s00335-018-9776-1 30151711 PMC6488303

[B15] Baxter V. K. Heise M. T. (2020). Immunopathogenesis of alphaviruses. Adv. Virus Res. 107, 315–382. doi: 10.1016/bs.aivir.2020.06.002 32711733 PMC8224468

[B16] Bellone R. Lechat P. Mousson L. Gilbart V. Piorkowski G. Bohers C.. (2023). Climate change and vector-borne diseases: a multi-omics approach of temperature-induced changes in the mosquito. J. Travel Med. 30 (4), taad062. doi: 10.1093/jtm/taad062 37171132

[B17] Bengue M. Ferraris P. Baronti C. Diagne C. T. Talignani L. Wichit S.. (2019). Mayaro virus infects human chondrocytes and induces the expression of arthritis-related genes associated with joint degradation. Viruses 11 (9), 797. doi: 10.3390/v11090797 31470617 PMC6783875

[B18] Bengue M. Ferraris P. Barthelemy J. Diagne C. T. Hamel R. Liegeois F.. (2021). Mayaro virus infects human brain cells and induces a potent antiviral response in human astrocytes. Viruses 13, 465. doi: 10.3390/v13030465 33799906 PMC8001792

[B19] Bocan T. M. Stafford R. G. Brown J. L. Akuoku Frimpong J. Basuli F. Hollidge B. S.. (2019). Characterization of brain inflammation, apoptosis, hypoxia, blood-brain barrier integrity and metabolism in Venezuelan equine encephalitis virus (VEEV TC-83) exposed mice by *in vivo* positron emission tomography imaging. Viruses 11 (11), 1052. doi: 10.3390/v11111052 31766138 PMC6893841

[B20] Bozza F. A. Moreira-Soto A. Rockstroh A. Fischer C. Nascimento A. D. Calheiros A. S.. (2019). Differential shedding and antibody kinetics of zika and chikungunya viruses, Brazil. Emerg. Infect. Dis. 25, 311–315. doi: 10.3201/eid2502.180166 30666934 PMC6346451

[B21] Brandstadter J. D. Yang Y. (2011). Natural killer cell responses to viral infection. J. Innate Immun. 3, 274–279. doi: 10.1159/000324176 21411975 PMC3128146

[B22] Bréhin A. C. Casadémont I. Frenkiel M. P. Julier C. Sakuntabhai A. Desprès P. (2009). The large form of human 2',5'-Oligoadenylate Synthetase (OAS3) exerts antiviral effect against Chikungunya virus. Virology 384, 216–222. doi: 10.1016/j.virol.2008.10.021 19056102

[B23] Broeckel R. Haese N. Messaoudi I. Streblow D. N. (2015). Nonhuman primate models of chikungunya virus infection and disease (CHIKV NHP model). Pathogens 4, 662–681. doi: 10.3390/pathogens4030662 26389957 PMC4584280

[B24] Brummer-Korvenkontio M. Vapalahti O. Kuusisto P. Saikku P. Manni T. Koskela P.. (2002). Epidemiology of Sindbis virus infections in Finland 1981-96: possible factors explaining a peculiar disease pattern. Epidemiol. Infect. 129, 335–345. doi: 10.1017/S0950268802007409 12403109 PMC2869892

[B25] Burke C. W. Gardner C. L. Steffan J. J. Ryman K. D. Klimstra W. B. (2009). Characteristics of alpha/beta interferon induction after infection of murine fibroblasts with wild-type and mutant alphaviruses. Virology 395, 121–132. doi: 10.1016/j.virol.2009.08.039 19782381 PMC4381786

[B26] Burt F. J. Chen W. Miner J. J. Lenschow D. J. Merits A. Schnettler E.. (2017). Chikungunya virus: an update on the biology and pathogenesis of this emerging pathogen. Lancet Infect. Dis. 17, e107–e117. doi: 10.1016/S1473-3099(16)30385-1 28159534

[B27] Cain M. D. Salimi H. Gong Y. Yang L. Hamilton S. L. Heffernan J. R.. (2017). Virus entry and replication in the brain precedes blood-brain barrier disruption during intranasal alphavirus infection. J. Neuroimmunol 308, 118–130. doi: 10.1016/j.jneuroim.2017.04.008 28501330 PMC5694394

[B28] Carpentier K. S. Morrison T. E. (2018). Innate immune control of alphavirus infection. Curr. Opin. Virol. 28, 53–60. doi: 10.1016/j.coviro.2017.11.006 29175515 PMC5835171

[B29] Cavalheiro M. G. Costa L. S. Campos H. S. Alves L. S. Assuncao-Miranda I. Poian A. T. (2016). Macrophages as target cells for Mayaro virus infection: involvement of reactive oxygen species in the inflammatory response during virus replication. Acad. Bras. Cienc 88, 1485–1499. doi: 10.1590/0001-3765201620150685 27627069

[B30] Cerny T. Schwarz M. Schwarz U. Lemant J. Gérardin P. Keller E. (2017). The range of neurological complications in chikungunya fever. Neurocrit Care 27, 447–457. doi: 10.1007/s12028-017-0413-8 28741102

[B31] Chan Y. H. Ng L. F. P. (2017). Age has a role in driving host immunopathological response to alphavirus infection. Immunology 152, 545–555. doi: 10.1111/imm.12799 28744856 PMC5680050

[B32] Charles P. C. Walters E. Margolis F. Johnston R. E. (1995). Mechanism of neuroinvasion of Venezuelan equine encephalitis virus in the mouse. Virology 208, 662–671. doi: 10.1006/viro.1995.1197 7747437

[B33] Chen W. Foo S. S. Sims N. A. Herrero L. J. Walsh N. C. Mahalingam S. (2015a). Arthritogenic alphaviruses: new insights into arthritis and bone pathology. Trends Microbiol. 23, 35–43. doi: 10.1016/j.tim.2014.09.005 25449049

[B34] Chen W. Foo S. S. Taylor A. Lulla A. Merits A. Hueston L.. (2015b). Bindarit, an inhibitor of monocyte chemotactic protein synthesis, protects against bone loss induced by chikungunya virus infection. J. Virol. 89, 581–593. doi: 10.1128/JVI.02034-14 25339772 PMC4301140

[B35] Chen I. Y. Ichinohe T. (2015). Response of host inflammasomes to viral infection. Trends Microbiol. 23, 55–63. doi: 10.1016/j.tim.2014.09.007 25456015

[B36] Chow A. Her Z. Ong E. K. Chen J. M. Dimatatac F. Kwek D. J.. (2011). Persistent arthralgia induced by Chikungunya virus infection is associated with interleukin-6 and granulocyte macrophage colony-stimulating factor. J. Infect. Dis. 203, 149–157. doi: 10.1093/infdis/jiq042 21288813 PMC3071069

[B37] Chua H. H. Abdul Rashid K. Law W. C. Hamizah A. Chem Y. K. Khairul A. H.. (2010). A fatal case of chikungunya virus infection with liver involvement. Med. J. Malaysia 65, 83–84. 21265260

[B38] Cirimotich C. M. Vela E. M. Garver J. Barnewall R. E. Miller B. D. Meister G. T.. (2017). Chikungunya virus infection in Cynomolgus macaques following Intradermal and aerosol exposure. Virol. J. 14, 135. doi: 10.1186/s12985-017-0804-7 28728590 PMC5520379

[B39] Cle M. Eldin P. Briant L. Lannuzel A. Simonin Y. Van de Perre P.. (2020). Neurocognitive impacts of arbovirus infections. J. Neuroinflamm. 17, 233. doi: 10.1186/s12974-020-01904-3 PMC7418199 32778106

[B40] Cook S. H. Griffin D. E. (2003). Luciferase imaging of a neurotropic viral infection in intact animals. J. Virol. 77, 5333–5338. doi: 10.1128/JVI.77.9.5333-5338.2003 12692235 PMC153972

[B41] Cook L. E. Locke M. C. Young A. R. Monte K. Hedberg M. L. Shimak R. M.. (2019). Distinct roles of interferon alpha and beta in controlling chikungunya virus replication and modulating neutrophil-mediated inflammation. J. Virol. 94 (1), e00841-19. doi: 10.1128/JVI.00841-19 31619554 PMC6912113

[B42] Couderc T. Chretien F. Schilte C. Disson O. Brigitte M. Guivel-Benhassine F.. (2008). A mouse model for Chikungunya: young age and inefficient type-I interferon signaling are risk factors for severe disease. PloS Pathog. 4, e29. doi: 10.1371/journal.ppat.0040029 18282093 PMC2242832

[B43] Couderc T. Khandoudi N. Grandadam M. Visse C. Gangneux N. Bagot S.. (2009). Prophylaxis and therapy for Chikungunya virus infection. J. Infect. Dis. 200, 516–523. doi: 10.1086/600381 19572805 PMC7109959

[B44] Danillo Lucas Alves E. Benedito Antonio Lopes da F. (2018). Characterization of the immune response following *in vitro* mayaro and chikungunya viruses (Alphavirus, Togaviridae) infection of mononuclear cells. Virus Res. 256, 166–173. doi: 10.1016/j.virusres.2018.08.011 30145137

[B45] Das T. Hoarau J. J. Bandjee M. C. J. Maquart M. Gasque P. (2015). Multifaceted innate immune responses engaged by astrocytes, microglia and resident dendritic cells against Chikungunya neuroinfection. J. Gen. Virol. 96, 294–310. doi: 10.1099/vir.0.071175-0 25351727

[B46] Davenport B. J. Bullock C. McCarthy M. K. Hawman D. W. Murphy K. M. Kedl R. M.. (2020). Chikungunya virus evades antiviral CD8(+) T cell responses to establish persistent infection in joint-associated tissues. J. Virol. 94 (9), e02036-19. doi: 10.1128/JVI.02036-19 32102875 PMC7163133

[B47] de Castro-Jorge L. A. de Carvalho R. V. H. Klein T. M. Hiroki C. H. Lopes A. H. Guimarães R. M.. (2019). The NLRP3 inflammasome is involved with the pathogenesis of Mayaro virus. PloS Pathog. 15, e1007934. doi: 10.1371/journal.ppat.1007934 31479495 PMC6743794

[B48] Decout A. Katz J. D. Venkatraman S. Ablasser A. (2021). The cGAS-STING pathway as a therapeutic target in inflammatory diseases. Nat. Rev. Immunol. 21, 548–569. doi: 10.1038/s41577-021-00524-z 33833439 PMC8029610

[B49] de França N. R. Mesquita Júnior D. Lima A. B. Pucci F. V. Andrade L. E. Silva N. P. (2010). RNA interference: a new alternative for rheumatic diseases therapy. Rev. Bras. Reumatol 50, 695–702. 21243308

[B50] Deresiewicz R. L. Thaler S. J. Hsu L. Zamani A. A. (1997). Clinical and neuroradiographic manifestations of eastern equine encephalitis. N Engl. J. Med. 336, 1867–1874. doi: 10.1056/NEJM199706263362604 9197215

[B51] de Souza W. M. Fumagalli M. J. de Lima S. T. S. Parise P. L. Carvalho D. C. M. Hernandez C.. (2024). Pathophysiology of chikungunya virus infection associated with fatal outcomes. Cell Host Microbe 32, 606–622.e608. doi: 10.1016/j.chom.2024.02.011 38479396 PMC11018361

[B52] Dias C. N. S. Gois B. M. Lima V. S. Guerra-Gomes I. C. Araujo J. M. G. Gomes J. A. S.. (2018). Human CD8 T-cell activation in acute and chronic chikungunya infection. Immunology 155, 499–504. doi: 10.1111/imm.12992 30099739 PMC6231013

[B53] Dropulić B. Masters C. L. (1990). Entry of neurotropic arboviruses into the central nervous system: an *in vitro* study using mouse brain endothelium. J. Infect. Dis. 161, 685–691. doi: 10.1093/infdis/161.4.685 2156944

[B54] Earnest J. T. Basore K. Roy V. Bailey A. L. Wang D. Alter G.. (2019). Neutralizing antibodies against Mayaro virus require Fc effector functions for protective activity. J. Exp. Med. 216, 2282–2301. doi: 10.1084/jem.20190736 31337735 PMC6781005

[B55] Economopoulou A. Dominguez M. Helynck B. Sissoko D. Wichmann O. Quenel P.. (2009). Atypical Chikungunya virus infections: clinical manifestations, mortality and risk factors for severe disease during the 2005-2006 outbreak on Réunion. Epidemiol. Infect. 137, 534–541. doi: 10.1017/S0950268808001167 18694529

[B56] Elrefaey A. M. E. Hollinghurst P. Reitmayer C. M. Alphey L. Maringer K. (2021). Innate immune antagonism of mosquito-borne flaviviruses in humans and mosquitoes. Viruses 13 (11), 2116. doi: 10.3390/v13112116 34834923 PMC8624719

[B57] Endy T. P. (2020). Viral febrile illnesses and emerging pathogens. Hunter's Trop. Med. Emerging Infect. Dis., 325–350. doi: 10.1016/B978-0-323-55512-8.00036-3

[B58] Fazakerley J. K. Boyd A. Mikkola M. L. Kääriäinen L. (2002). A single amino acid change in the nuclear localization sequence of the nsP2 protein affects the neurovirulence of Semliki Forest virus. J. Virol. 76, 392–396. doi: 10.1128/JVI.76.1.392-396.2002 11739703 PMC135702

[B59] Ferraris P. Cochet M. Hamel R. Gladwyn-Ng I. Alfano C. Diop F.. (2019). Zika virus differentially infects human neural progenitor cells according to their state of differentiation and dysregulates neurogenesis through the Notch pathway. Emerg. Microbes Infect. 8, 1003–1016. doi: 10.1080/22221751.2019.1637283 31282298 PMC6691766

[B60] Figueiredo C. M. Neris R. Gavino-Leopoldino D. da Silva M. O. L. Almeida J. S. Dos-Santos J. S.. (2019). Mayaro virus replication restriction and induction of muscular inflammation in mice are dependent on age, type-I interferon response, and adaptive immunity. Front. Microbiol. 10. doi: 10.3389/fmicb.2019.02246 PMC6779782 31632368

[B61] Fox J. M. Long F. Edeling M. A. Lin H. van Duijl-Richter M. K. S. Fong R. H.. (2015). Broadly neutralizing alphavirus antibodies bind an epitope on E2 and inhibit entry and egress. Cell 163, 1095–1107. doi: 10.1016/j.cell.2015.10.050 26553503 PMC4659373

[B62] Fraser J. R. Becker G. J. (1984). Mononuclear cell types in chronic synovial effusions of Ross River virus disease. Aust. N Z J. Med. 14, 505–506. doi: 10.1111/j.1445-5994.1984.tb03629.x 6097207

[B63] Frolov I. Akhrymuk M. Akhrymuk I. Atasheva S. Frolova E. I. (2012). Early events in alphavirus replication determine the outcome of infection. J. Virol. 86, 5055–5066. doi: 10.1128/JVI.07223-11 22345447 PMC3347369

[B64] Fros J. J. Liu W. J. Prow N. A. Geertsema C. Ligtenberg M. Vanlandingham D. L.. (2010). Chikungunya virus nonstructural protein 2 inhibits type I/II interferon-stimulated JAK-STAT signaling. J. Virol. 84, 10877–10887. doi: 10.1128/JVI.00949-10 20686047 PMC2950581

[B65] Fros J. J. Pijlman G. P. (2016). Alphavirus infection: host cell shut-off and inhibition of antiviral responses. Viruses 8 (6), 166. doi: 10.3390/v8060166 27294951 PMC4926186

[B66] Fros J. J. van der Maten E. Vlak J. M. Pijlman G. P. (2013). The C-terminal domain of chikungunya virus nsP2 independently governs viral RNA replication, cytopathicity, and inhibition of interferon signaling. J. Virol. 87, 10394–10400. doi: 10.1128/JVI.00884-13 23864632 PMC3753987

[B67] Ganu M. A. Ganu A. S. (2011). Post-chikungunya chronic arthritis–our experience with DMARDs over two year follow up. J. Assoc. Physicians India 59, 83–86. 21751641

[B68] Gardner J. Anraku I. Le T. T. Larcher T. Major L. Roques P.. (2010). Chikungunya virus arthritis in adult wild-type mice. J. Virol. 84, 8021–8032. doi: 10.1128/JVI.02603-09 20519386 PMC2916516

[B69] Gardner C. L. Burke C. W. Higgs S. T. Klimstra W. B. Ryman K. D. (2012). Interferon-alpha/beta deficiency greatly exacerbates arthritogenic disease in mice infected with wild-type chikungunya virus but not with the cell culture-adapted live-attenuated 181/25 vaccine candidate. Virology 425, 103–112. doi: 10.1016/j.virol.2011.12.020 22305131 PMC3431213

[B70] Gardner C. L. Burke C. W. Tesfay M. Z. Glass P. J. Klimstra W. B. Ryman K. D. (2008). Eastern and Venezuelan equine encephalitis viruses differ in their ability to infect dendritic cells and macrophages: impact of altered cell tropism on pathogenesis. J. Virol. 82, 10634–10646. doi: 10.1128/JVI.01323-08 18768986 PMC2573165

[B71] Gardner J. P. Frolov I. Perri S. Ji Y. MacKichan M. L. zur Megede J.. (2000). Infection of human dendritic cells by a sindbis virus replicon vector is determined by a single amino acid substitution in the E2 glycoprotein. J. Virol. 74, 11849–11857. doi: 10.1128/JVI.74.24.11849-11857.2000 11090185 PMC112468

[B72] Garmashova N. Gorchakov R. Frolova E. Frolov I. (2006). Sindbis virus nonstructural protein nsP2 is cytotoxic and inhibits cellular transcription. J. Virol. 80, 5686–5696. doi: 10.1128/JVI.02739-05 16731907 PMC1472573

[B73] Garmashova N. Gorchakov R. Volkova E. Paessler S. Frolova E. Frolov I. (2007). The Old World and New World alphaviruses use different virus-specific proteins for induction of transcriptional shutoff. J. Virol. 81, 2472–2484. doi: 10.1128/JVI.02073-06 17108023 PMC1865960

[B74] Gasque P. Couderc T. Lecuit M. Roques P. Ng L. F. (2015). Chikungunya virus pathogenesis and immunity. Vector Borne Zoonotic Dis. 15, 241–249. doi: 10.1089/vbz.2014.1710 25897810

[B75] Gauri L. A. Ranwa B. L. Nagar K. Vyas A. Fatima Q. (2012). Post chikungunya brain stem encephalitis. J. Assoc. Physicians India 60, 68–70. 23029750

[B76] Gerardin P. Barau G. Michault A. Bintner M. Randrianaivo H. Choker G.. (2008). Multidisciplinary prospective study of mother-to-child chikungunya virus infections on the island of La Reunion. PloS Med. 5, e60. doi: 10.1371/journal.pmed.0050060 18351797 PMC2267812

[B77] Gérardin P. Sampériz S. Ramful D. Boumahni B. Bintner M. Alessandri J. L.. (2014). Neurocognitive outcome of children exposed to perinatal mother-to-child Chikungunya virus infection: the CHIMERE cohort study on Reunion Island. PloS Negl. Trop. Dis. 8, e2996. doi: 10.1371/journal.pntd.0002996 25033077 PMC4102444

[B78] Ghosh S. Larson S. D. Hefzi H. Marnoy Z. Cutforth T. Dokka K.. (2011). Sensory maps in the olfactory cortex defined by long-range viral tracing of single neurons. Nature 472, 217–220. doi: 10.1038/nature09945 21451523

[B79] Gleiser C. A. Gochenour W. S. Jr. Berge T. O. Tigertt W. D. (1962). The comparative pathology of experimental Venezuelan equine encephalomyelitis infection in different animal hosts. J. Infect. Dis. 110, 80–97. doi: 10.1093/infdis/110.1.80 13899188

[B80] Göertz G. P. McNally K. L. Robertson S. J. Best S. M. Pijlman G. P. Fros J. J. (2018). The methyltransferase-like domain of chikungunya virus nsP2 inhibits the interferon response by promoting the nuclear export of STAT1. J. Virol. 92 (17), e01008-18. doi: 10.1128/JVI.01008-18 29925658 PMC6096799

[B81] Gorelkin L. (1973). Venezuelan equine encephalomyelitis in an adult animal host. An electron microscopic study. Am. J. Pathol. 73, 425–442. 4796626 PMC1904063

[B82] Goupil B. A. Mores C. N. (2016). A review of chikungunya virus-induced arthralgia: clinical manifestations, therapeutics, and pathogenesis. Open Rheumatol J. 10, 129–140. doi: 10.2174/1874312901610010129 28077980 PMC5204064

[B83] Grieder F. B. Vogel S. N. (1999). Role of interferon and interferon regulatory factors in early protection against Venezuelan equine encephalitis virus infection. Virology 257, 106–118. doi: 10.1006/viro.1999.9662 10208925

[B84] Griffin D. E. (1989). Molecular pathogenesis of Sindbis virus encephalitis in experimental animals. Adv. Virus Res. 36, 255–271. doi: 10.1016/S0065-3527(08)60587-4 2544083

[B85] Griffin D. E. (1992). “Alphaviruses, flaviviruses, and bunyaviruses,” in Neuropathogenic viruses and immunity. Eds. Specter S. Bendinelli M. Friedman H. (Springer US, Boston, MA), 255–274.

[B86] Griffin D. E. (2005). Neuronal cell death in alphavirus encephalomyelitis. Curr. Top. Microbiol. Immunol. 289, 57–77. doi: 10.1007/b138916 15791951

[B87] Guerrero-Arguero I. Tellez-Freitas C. M. Weber K. S. Berges B. K. Robison R. A. Pickett B. E. (2021). Alphaviruses: Host pathogenesis, immune response, and vaccine & treatment updates. J. Gen. Virol. 102 (8). doi: 10.1099/jgv.0.001644 34435944

[B88] Gunn B. M. Jones J. E. Shabman R. S. Whitmore A. C. Sarkar S. Blevins L. K.. (2018). Ross River virus envelope glycans contribute to disease through activation of the host complement system. Virology 515, 250–260. doi: 10.1016/j.virol.2017.12.022 29324290 PMC7119116

[B89] Gunn B. M. Morrison T. E. Whitmore A. C. Blevins L. K. Hueston L. Fraser R. J.. (2012). Mannose binding lectin is required for alphavirus-induced arthritis/myositis. PloS Pathog. 8, e1002586. doi: 10.1371/journal.ppat.1002586 22457620 PMC3310795

[B90] Gupta P. Sharma A. Han J. Yang A. Bhomia M. Knollmann-Ritschel B.. (2017). Differential host gene responses from infection with neurovirulent and partially-neurovirulent strains of Venezuelan equine encephalitis virus. BMC Infect. Dis. 17, 309. doi: 10.1186/s12879-017-2355-3 28446152 PMC5405508

[B91] Harley D. Sleigh A. Ritchie S. (2001). Ross River virus transmission, infection, and disease: a cross-disciplinary review. Clin. Microbiol. Rev. 14, 909–932. doi: 10.1128/CMR.14.4.909-932.2001 11585790 PMC89008

[B92] Hawman D. W. Stoermer K. A. Montgomery S. A. Pal P. Oko L. Diamond M. S.. (2013). Chronic joint disease caused by persistent Chikungunya virus infection is controlled by the adaptive immune response. J. Virol. 87, 13878–13888. doi: 10.1128/JVI.02666-13 24131709 PMC3838294

[B93] Heise M. T. Simpson D. A. Johnston R. E. (2000). Sindbis-group alphavirus replication in periosteum and endosteum of long bones in adult mice. J. Virol. 74, 9294–9299. doi: 10.1128/JVI.74.19.9294-9299.2000 10982376 PMC102128

[B94] Her Z. Malleret B. Chan M. Ong E. K. Wong S. C. Kwek D. J.. (2010). Active infection of human blood monocytes by Chikungunya virus triggers an innate immune response. J. Immunol. 184, 5903–5913. doi: 10.4049/jimmunol.0904181 20404274

[B95] Hertzog P. Forster S. Samarajiwa S. (2011). Systems biology of interferon responses. J. Interferon Cytokine Res. 31, 5–11. doi: 10.1089/jir.2010.0126 21226606

[B96] Hibl B. M. Dailey Garnes N. J. M. Kneubehl A. R. Vogt M. B. Spencer Clinton J. L. Rico-Hesse R. R. (2021). Mosquito-bite infection of humanized mice with chikungunya virus produces systemic disease with long-term effects. PloS Negl. Trop. Dis. 15, e0009427. doi: 10.1371/journal.pntd.0009427 34106915 PMC8189471

[B97] Hoarau J. J. Gay F. Pelle O. Samri A. Jaffar-Bandjee M. C. Gasque P.. (2013). Identical strength of the T cell responses against E2, nsP1 and capsid CHIKV proteins in recovered and chronic patients after the epidemics of 2005-2006 in La Reunion Island. PloS One 8, e84695. doi: 10.1371/journal.pone.0084695 24376836 PMC3871564

[B98] Hoarau J. J. Jaffar Bandjee M. C. Krejbich Trotot P. Das T. Li-Pat-Yuen G. Dassa B.. (2010). Persistent chronic inflammation and infection by Chikungunya arthritogenic alphavirus in spite of a robust host immune response. J. Immunol. 184, 5914–5927. doi: 10.4049/jimmunol.0900255 20404278

[B99] Honnold S. P. Mossel E. C. Bakken R. R. Lind C. M. Cohen J. W. Eccleston L. T.. (2015). Eastern equine encephalitis virus in mice II: pathogenesis is dependent on route of exposure. Virol. J. 12, 154. doi: 10.1186/s12985-015-0385-2 26423229 PMC4589026

[B100] Hoz J. M. Bayona B. Viloria S. Accini J. L. Juan-Vergara H. S. Viasus D. (2015). Fatal cases of Chikungunya virus infection in Colombia: Diagnostic and treatment challenges. J. Clin. Virol. 69, 27–29. doi: 10.1016/j.jcv.2015.05.021 26209372

[B101] Hu X. Li J. Fu M. Zhao X. Wang W. (2021). The JAK/STAT signaling pathway: from bench to clinic. Signal Transduct Target Ther. 6, 402. doi: 10.1038/s41392-021-00791-1 34824210 PMC8617206

[B102] Inglis F. M. Lee K. M. Chiu K. B. Purcell O. M. Didier P. J. Russell-Lodrigue K.. (2016). Neuropathogenesis of Chikungunya infection: astrogliosis and innate immune activation. J. Neurovirol 22, 140–148. doi: 10.1007/s13365-015-0378-3 26419894 PMC4783292

[B103] Ishida R. Cole J. Lopez-Orozco J. Fayad N. Felix-Lopez A. Elaish M.. (2021). Mayaro virus non-structural protein 2 circumvents the induction of interferon in part by depleting host transcription initiation factor IIE subunit 2. Cells 10 (12), 3510. doi: 10.3390/cells10123510 34944018 PMC8700540

[B104] Jaffar-Bandjee M. C. Das T. Hoarau J. J. Krejbich Trotot P. Denizot M. Ribera A.. (2009). Chikungunya virus takes centre stage in virally induced arthritis: possible cellular and molecular mechanisms to pathogenesis. Microbes Infect. 11, 1206–1218. doi: 10.1016/j.micinf.2009.10.001 19835977

[B105] Jenster L. M. Lange K. E. Normann S. vom Hemdt A. Wuerth J. D. Schiffelers L. D. J.. (2023). P38 kinases mediate NLRP1 inflammasome activation after ribotoxic stress response and virus infection. J. Exp. Med. 220 (1), e20220837. doi: 10.1084/jem.20220837 36315050 PMC9623368

[B106] Jin J. Liss N. M. Chen D. H. Liao M. Fox J. M. Shimak R. M.. (2015). Neutralizing monoclonal antibodies block chikungunya virus entry and release by targeting an epitope critical to viral pathogenesis. Cell Rep. 13, 2553–2564. doi: 10.1016/j.celrep.2015.11.043 26686638 PMC4720387

[B107] Kafai N. M. Diamond M. S. Fox J. M. (2022). Distinct cellular tropism and immune responses to alphavirus infection. Annu. Rev. Immunol. 40, 615–649. doi: 10.1146/annurev-immunol-101220-014952 35134315 PMC10350340

[B108] Kam Y. W. Lee W. W. Simarmata D. Harjanto S. Teng T. S. Tolou H.. (2012a). Longitudinal analysis of the human antibody response to Chikungunya virus infection: implications for serodiagnosis and vaccine development. J. Virol. 86, 13005–13015. doi: 10.1128/JVI.01780-12 23015702 PMC3497641

[B109] Kam Y. W. Lum F. M. Teo T. H. Lee W. W. Simarmata D. Harjanto S.. (2012b). Early neutralizing IgG response to Chikungunya virus in infected patients targets a dominant linear epitope on the E2 glycoprotein. EMBO Mol. Med. 4, 330–343. doi: 10.1002/emmm.201200213 22389221 PMC3376860

[B110] Kam Y. W. Simarmata D. Chow A. Her Z. Teng T. S. Ong E. K.. (2012c). Early appearance of neutralizing immunoglobulin G3 antibodies is associated with chikungunya virus clearance and long-term clinical protection. J. Infect. Dis. 205, 1147–1154. doi: 10.1093/infdis/jis033 22389226 PMC3295607

[B111] Kashyap R. S. Morey S. Bhullar S. Baheti N. Chandak N. Purohit H.. (2014). Determination of Toll-like receptor-induced cytokine profiles in the blood and cerebrospinal fluid of Chikungunya patients. Neuroimmunomodulation 21, 338–346. doi: 10.1159/000358240 24776821

[B112] Katze M. G. He Y. Gale M. Jr (2002). Viruses and interferon: a fight for supremacy. Nat. Rev. Immunol. 2, 675–687. doi: 10.1038/nri888 12209136

[B113] Kim A. S. Diamond M. S. (2023). A molecular understanding of alphavirus entry and antibody protection. Nat. Rev. Microbiol. 21, 396–407. doi: 10.1038/s41579-022-00825-7 36474012 PMC9734810

[B114] Kolliker-Frers R. Udovin L. Otero-Losada M. Kobiec T. Herrera M. I. Palacios J.. (2021). Neuroinflammation: an integrating overview of reactive-neuroimmune cell interactions in health and disease. Mediators Inflammation 2021, 9999146. doi: 10.1155/2021/9999146 PMC8187052 34158806

[B115] Kulcsar K. A. Baxter V. K. Greene I. P. Griffin D. E. (2014). Interleukin 10 modulation of pathogenic Th17 cells during fatal alphavirus encephalomyelitis. Proc. Natl. Acad. Sci. U.S.A. 111, 16053–16058. doi: 10.1073/pnas.1418966111 25362048 PMC4234572

[B116] Kulcsar K. A. Griffin D. E. (2016). T cell-derived interleukin-10 is an important regulator of the Th17 response during lethal alphavirus encephalomyelitis. J. Neuroimmunol 295-296, 60–67. doi: 10.1016/j.jneuroim.2016.04.010 27235350 PMC4884611

[B117] Kutchko K. M. Madden E. A. Morrison C. Plante K. S. Sanders W. Vincent H. A.. (2018). Structural divergence creates new functional features in alphavirus genomes. Nucleic Acids Res. 46, 3657–3670. doi: 10.1093/nar/gky012 29361131 PMC6283419

[B118] Labadie K. Larcher T. Joubert C. Mannioui A. Delache B. Brochard P.. (2010). Chikungunya disease in nonhuman primates involves long-term viral persistence in macrophages. J. Clin. Invest. 120, 894–906. doi: 10.1172/JCI40104 20179353 PMC2827953

[B119] Landers V. D. Wilkey D. W. Merchant M. L. Mitchell T. C. Sokoloski K. J. (2021). The alphaviral capsid protein inhibits IRAK1-dependent TLR signaling. Viruses 13 (3), 377. doi: 10.3390/v13030377 33673546 PMC7997285

[B120] Lazear H. M. Schoggins J. W. Diamond M. S. (2019). Shared and distinct functions of type I and type III interferons. Immunity 50, 907–923. doi: 10.1016/j.immuni.2019.03.025 30995506 PMC6839410

[B121] Lebrun G. Chadda K. Reboux A. H. Martinet O. Gauzere B. A. (2009). Guillain-Barre syndrome after chikungunya infection. Emerg. Infect. Dis. 15, 495–496. doi: 10.3201/eid1503.071482 19239775 PMC2681104

[B122] Lee S. H. Miyagi T. Biron C. A. (2007). Keeping NK cells in highly regulated antiviral warfare. Trends Immunol. 28, 252–259. doi: 10.1016/j.it.2007.04.001 17466596

[B123] Lenschow D. J. Lai C. Frias-Staheli N. Giannakopoulos N. V. Lutz A. Wolff T.. (2007). IFN-stimulated gene 15 functions as a critical antiviral molecule against influenza, herpes, and Sindbis viruses. Proc. Natl. Acad. Sci. U.S.A. 104, 1371–1376. doi: 10.1073/pnas.0607038104 17227866 PMC1783119

[B124] Li W. X. Ding S. W. (2022). Mammalian viral suppressors of RNA interference. Trends Biochem. Sci. 47, 978–988. doi: 10.1016/j.tibs.2022.05.001 35618579 PMC10281742

[B125] Liang Q. Luo Z. Zeng J. Chen W. Foo S. S. Lee S. A.. (2016). Zika virus NS4A and NS4B proteins deregulate akt-mTOR signaling in human fetal neural stem cells to inhibit neurogenesis and induce autophagy. Cell Stem Cell 19, 663–671. doi: 10.1016/j.stem.2016.07.019 27524440 PMC5144538

[B126] Lidbury B. A. Rulli N. E. Suhrbier A. Smith P. N. McColl S. R. Cunningham A. L.. (2008). Macrophage-derived proinflammatory factors contribute to the development of arthritis and myositis after infection with an arthrogenic alphavirus. J. Infect. Dis. 197, 1585–1593. doi: 10.1086/587841 18433328

[B127] Lidbury B. A. Simeonovic C. Maxwell G. E. Marshall I. D. Hapel A. J. (2000). Macrophage-induced muscle pathology results in morbidity and mortality for Ross River virus-infected mice. J. Infect. Dis. 181, 27–34. doi: 10.1086/315164 10608747

[B128] Lindsey N. P. Staples J. E. Fischer M. (2018). Eastern equine encephalitis virus in the United States 2003-2016. Am. J. Trop. Med. Hyg 98, 1472–1477. doi: 10.4269/ajtmh.17-0927 29557336 PMC5953388

[B129] Linn M. L. Mateo L. Gardner J. Suhrbier A. (1998). Alphavirus-specific cytotoxic T lymphocytes recognize a cross-reactive epitope from the capsid protein and can eliminate virus from persistently infected macrophages. J. Virol. 72, 5146–5153. doi: 10.1128/JVI.72.6.5146-5153.1998 9573286 PMC110085

[B130] Liu X. Poo Y. S. Alves J. C. Almeida R. P. Mostafavi H. Tang P. C. H.. (2022a). Interleukin-17 contributes to chikungunya virus-induced disease. mBio 13, e0028922. doi: 10.1128/mbio.00289-22 35254128 PMC9040832

[B131] Liu J. L. Shriver-Lake L. C. Zabetakis D. Goldman E. R. Anderson G. P. (2018). Selection of single-domain antibodies towards western equine encephalitis virus. Antibodies (Basel) 7 (4), 44. doi: 10.3390/antib7040044 31544894 PMC6698954

[B132] Liu Y. Yuan Y. Zhang L. (2022b). Innate immune evasion by alphaviruses. Front. Immunol. 13. doi: 10.3389/fimmu.2022.1005586 PMC9510981 36172361

[B133] Logue C. H. Bosio C. F. Welte T. Keene K. M. Ledermann J. P. Phillips A.. (2009). Virulence variation among isolates of western equine encephalitis virus in an outbred mouse model. J. Gen. Virol. 90, 1848–1858. doi: 10.1099/vir.0.008656-0 19403754 PMC2887574

[B134] Long K. M. Whitmore A. C. Ferris M. T. Sempowski G. D. McGee C. Trollinger B.. (2013). Dendritic cell immunoreceptor regulates Chikungunya virus pathogenesis in mice. J. Virol. 87, 5697–5706. doi: 10.1128/JVI.01611-12 23487448 PMC3648201

[B135] Malvy D. Ezzedine K. Mamani-Matsuda M. Autran B. Tolou H. Receveur M. C.. (2009). Destructive arthritis in a patient with chikungunya virus infection with persistent specific IgM antibodies. BMC Infect. Dis. 9, 200. doi: 10.1186/1471-2334-9-200 20003320 PMC2803790

[B136] Manimunda S. P. Vijayachari P. Uppoor R. Sugunan A. P. Singh S. S. Rai S. K.. (2010). Clinical progression of chikungunya fever during acute and chronic arthritic stages and the changes in joint morphology as revealed by imaging. Trans. R Soc. Trop. Med. Hyg 104, 392–399. doi: 10.1016/j.trstmh.2010.01.011 20171708

[B137] Maucourant C. Petitdemange C. Yssel H. Vieillard V. (2019). Control of acute arboviral infection by natural killer cells. Viruses 11 (2), 131. doi: 10.3390/v11020131 30709036 PMC6410043

[B138] McKinley M. J. McAllen R. M. Davern P. Giles M. E. Penschow J. Sunn N.. (2003). The sensory circumventricular organs of the mammalian brain. Adv. Anat Embryol Cell Biol. 172, 1–122. doi: 10.1007/978-3-642-55532-9. III-XII. 12901335

[B139] Mehta R. Gerardin P. de Brito C. A. A. Soares C. N. Ferreira M. L. B. Solomon T. (2018). The neurological complications of chikungunya virus: A systematic review. Rev. Med. Virol. 28, e1978. doi: 10.1002/rmv.1978 29671914 PMC5969245

[B140] Mercado M. Acosta-Reyes J. Parra E. Guzman L. Beltran M. Gasque P.. (2018). Renal involvement in fatal cases of chikungunya virus infection. J. Clin. Virol. 103, 16–18. doi: 10.1016/j.jcv.2018.03.009 29604514

[B141] Mercado M. Acosta-Reyes J. Parra E. Pardo L. Rico A. Campo A.. (2016). Clinical and histopathological features of fatal cases with dengue and chikungunya virus co-infection in Colombia 2014 to 2015. Euro Surveill 21 (22). doi: 10.2807/1560-7917.ES.2016.21.22.30244 27277216

[B142] Miner J. J. Aw-Yeang H. X. Fox J. M. Taffner S. Malkova O. N. Oh S. T.. (2015). Chikungunya viral arthritis in the United States: a mimic of seronegative rheumatoid arthritis. Arthritis Rheumatol 67, 1214–1220. doi: 10.1002/art.39027 25605621 PMC4591551

[B143] Miner J. J. Cook L. E. Hong J. P. Smith A. M. Richner J. M. Shimak R. M.. (2017). Therapy with CTLA4-Ig and an antiviral monoclonal antibody controls chikungunya virus arthritis. Sci. Transl. Med. 9 (375), eaah3438. doi: 10.1126/scitranslmed.aah3438 28148840 PMC5448557

[B144] Mittal A. Mittal S. Bharati M. J. Ramakrishnan R. Saravanan S. Sathe P. S. (2007). Optic neuritis associated with chikungunya virus infection in South India. Arch. Ophthalmol. 125, 1381–1386. doi: 10.1001/archopht.125.10.1381 17923547

[B145] Miyata S. (2015). New aspects in fenestrated capillary and tissue dynamics in the sensory circumventricular organs of adult brains. Front. Neurosci. 9. doi: 10.3389/fnins.2015.00390 PMC4621430 26578857

[B146] Morens D. M. Fauci A. S. (2012). Emerging infectious diseases in 2012: 20 years after the institute of medicine report. mBio 3 (6), e00494-12. doi: 10.1128/mBio.00494-12 23232716 PMC3520107

[B147] Morita S. Miyata S. (2012). Different vascular permeability between the sensory and secretory circumventricular organs of adult mouse brain. Cell Tissue Res. 349, 589–603. doi: 10.1007/s00441-012-1421-9 22584508

[B148] Morrison T. E. Fraser R. J. Smith P. N. Mahalingam S. Heise M. T. (2007). Complement contributes to inflammatory tissue destruction in a mouse model of Ross River virus-induced disease. J. Virol. 81, 5132–5143. doi: 10.1128/JVI.02799-06 17314163 PMC1900244

[B149] Morrison T. E. Oko L. Montgomery S. A. Whitmore A. C. Lotstein A. R. Gunn B. M.. (2011). A mouse model of chikungunya virus-induced musculoskeletal inflammatory disease: evidence of arthritis, tenosynovitis, myositis, and persistence. Am. J. Pathol. 178, 32–40. doi: 10.1016/j.ajpath.2010.11.018 21224040 PMC3069999

[B150] Morrison T. E. Simmons J. D. Heise M. T. (2008). Complement receptor 3 promotes severe ross river virus-induced disease. J. Virol. 82, 11263–11272. doi: 10.1128/JVI.01352-08 18787004 PMC2573283

[B151] Morrison T. E. Whitmore A. C. Shabman R. S. Lidbury B. A. Mahalingam S. Heise M. T. (2006). Characterization of Ross River virus tropism and virus-induced inflammation in a mouse model of viral arthritis and myositis. J. Virol. 80, 737–749. doi: 10.1128/JVI.80.2.737-749.2006 16378976 PMC1346871

[B152] Mostafavi H. Abeyratne E. Zaid A. Taylor A. (2019). Arthritogenic alphavirus-induced immunopathology and targeting host inflammation as A therapeutic strategy for alphaviral disease. Viruses 11 (3), 290. doi: 10.3390/v11030290 30909385 PMC6466158

[B153] Mostafavi H. Tharmarajah K. Vider J. West N. P. Freitas J. R. Cameron B.. (2022). Interleukin-17 contributes to Ross River virus-induced arthritis and myositis. PloS Pathog. 18, e1010185. doi: 10.1371/journal.ppat.1010185 35143591 PMC8830676

[B154] Murira A. Lamarre A. (2016). Type-I interferon responses: from friend to foe in the battle against chronic viral infection. Front. Immunol. 7. doi: 10.3389/fimmu.2016.00609 PMC5165262 28066419

[B155] Neighbours L. M. Long K. Whitmore A. C. Heise M. T. (2012). Myd88-dependent toll-like receptor 7 signaling mediates protection from severe Ross River virus-induced disease in mice. J. Virol. 86, 10675–10685. doi: 10.1128/JVI.00601-12 22837203 PMC3457316

[B156] Ng L. F. Chow A. Sun Y. J. Kwek D. J. Lim P. L. Dimatatac F.. (2009). IL-1beta, IL-6, and RANTES as biomarkers of Chikungunya severity. PloS One 4, e4261. doi: 10.1371/journal.pone.0004261 19156204 PMC2625438

[B157] Ng L. F. P. Renia L. (2024). Live-attenuated chikungunya virus vaccine. Cell 187, 813–813.e811. doi: 10.1016/j.cell.2024.01.033 38364787

[B158] Nimmannitya S. Halstead S. B. Cohen S. N. Margiotta M. R. (1969). Dengue and chikungunya virus infection in man in Thailand 1962-1964. I. Observations on hospitalized patients with hemorrhagic fever. Am. J. Trop. Med. Hyg 18, 954–971. doi: 10.4269/ajtmh.1969.18.954 5355242

[B159] Nitatpattana N. Kanjanopas K. Yoksan S. Satimai W. Vongba N. Langdatsuwan S.. (2014). Long-term persistence of Chikungunya virus neutralizing antibodies in human populations of North Eastern Thailand. Virol. J. 11, 183. doi: 10.1186/1743-422X-11-183 25330992 PMC4283153

[B160] Ong R.-Y. Lum F.-M. Ng L. F. (2014). The fine line between protection and pathology in neurotropic flavivirus and alphavirus infections. Future Virology 9, 3, 313–330. doi: 10.2217/fvl.14.6

[B161] Ozden S. Huerre M. Riviere J. P. Coffey L. L. Afonso P. V. Mouly V.. (2007). Human muscle satellite cells as targets of Chikungunya virus infection. PloS One 2, e527. doi: 10.1371/journal.pone.0000527 17565380 PMC1885285

[B162] Pakran J. George M. Riyaz N. Arakkal R. George S. Rajan U.. (2011). Purpuric macules with vesiculobullous lesions: a novel manifestation of Chikungunya. Int. J. Dermatol. 50, 61–69. doi: 10.1111/j.1365-4632.2010.04644.x 21182504

[B163] Pal P. Dowd K. A. Brien J. D. Edeling M. A. Gorlatov S. Johnson S.. (2013). Development of a highly protective combination monoclonal antibody therapy against Chikungunya virus. PloS Pathog. 9, e1003312. doi: 10.1371/journal.ppat.1003312 23637602 PMC3630103

[B164] Pal P. Fox J. M. Hawman D. W. Huang Y. J. Messaoudi I. Kreklywich C.. (2014). Chikungunya viruses that escape monoclonal antibody therapy are clinically attenuated, stable, and not purified in mosquitoes. J. Virol. 88, 8213–8226. doi: 10.1128/JVI.01032-14 24829346 PMC4135940

[B165] Partidos C. D. Paykel J. Weger J. Borland E. M. Powers A. M. Seymour R.. (2012). Cross-protective immunity against o'nyong-nyong virus afforded by a novel recombinant chikungunya vaccine. Vaccine 30, 4638–4643. doi: 10.1016/j.vaccine.2012.04.099 22583812 PMC3372665

[B166] Paul S. D. Singh K. R. (1968). Experimental infection of Macaca radiata with Chikungunya virus and transmission of virus by mosquitoes. Indian J. Med. Res. 56, 802–811. 4971384

[B167] Petitdemange C. Becquart P. Wauquier N. Beziat V. Debre P. Leroy E. M.. (2011). Unconventional repertoire profile is imprinted during acute chikungunya infection for natural killer cells polarization toward cytotoxicity. PloS Pathog. 7, e1002268. doi: 10.1371/journal.ppat.1002268 21966274 PMC3178577

[B168] Petitdemange C. Wauquier N. Vieillard V. (2015). Control of immunopathology during chikungunya virus infection. J. Allergy Clin. Immunol. 135, 846–855. doi: 10.1016/j.jaci.2015.01.039 25843597

[B169] Petrakova O. Volkova E. Gorchakov R. Paessler S. Kinney R. M. Frolov I. (2005). Noncytopathic replication of Venezuelan equine encephalitis virus and eastern equine encephalitis virus replicons in Mammalian cells. J. Virol. 79, 7597–7608. doi: 10.1128/JVI.79.12.7597-7608.2005 15919912 PMC1143662

[B170] Phelps A. L. O'Brien L. M. Eastaugh L. S. Davies C. Lever M. S. Ennis J.. (2017). Susceptibility and lethality of western equine encephalitis virus in balb/c mice when infected by the aerosol route. Viruses 9 (7), 163. doi: 10.3390/v9070163 28654007 PMC5537655

[B171] Phillips A. T. Rico A. B. Stauft C. B. Hammond S. L. Aboellail T. A. Tjalkens R. B.. (2016). Entry sites of Venezuelan and western equine encephalitis viruses in the mouse central nervous system following peripheral infection. J. Virol. 90, 5785–5796. doi: 10.1128/JVI.03219-15 27053560 PMC4886771

[B172] Phillips A. T. Stauft C. B. Aboellail T. A. Toth A. M. Jarvis D. L. Powers A. M.. (2013). Bioluminescent imaging and histopathologic characterization of WEEV neuroinvasion in outbred CD-1 mice. PloS One 8, e53462. doi: 10.1371/journal.pone.0053462 23301074 PMC3534643

[B173] Phuklia W. Kasisith J. Modhiran N. Rodpai E. Thannagith M. Thongsakulprasert T.. (2013). Osteoclastogenesis induced by CHIKV-infected fibroblast-like synoviocytes: a possible interplay between synoviocytes and monocytes/macrophages in CHIKV-induced arthralgia/arthritis. Virus Res. 177, 179–188. doi: 10.1016/j.virusres.2013.08.011 24012515

[B174] Pierro A. Rossini G. Gaibani P. Finarelli A. C. Moro M. L. Landini M. P.. (2015). Persistence of anti-chikungunya virus-specific antibodies in a cohort of patients followed from the acute phase of infection after the 2007 outbreak in Italy. New Microbes New Infect. 7, 23–25. doi: 10.1016/j.nmni.2015.04.002 26106482 PMC4475829

[B175] Poo Y. S. Nakaya H. Gardner J. Larcher T. Schroder W. A. Le T. T.. (2014a). CCR2 deficiency promotes exacerbated chronic erosive neutrophil-dominated chikungunya virus arthritis. J. Virol. 88, 6862–6872. doi: 10.1128/JVI.03364-13 24696480 PMC4054367

[B176] Poo Y. S. Rudd P. A. Gardner J. Wilson J. A. Larcher T. Colle M. A.. (2014b). Multiple immune factors are involved in controlling acute and chronic chikungunya virus infection. PloS Negl. Trop. Dis. 8, e3354. doi: 10.1371/journal.pntd.0003354 25474568 PMC4256279

[B177] Powell L. A. Fox J. M. Kose N. Kim A. S. Majedi M. Bombardi R.. (2020). Human monoclonal antibodies against Ross River virus target epitopes within the E2 protein and protect against disease. PloS Pathog. 16, e1008517. doi: 10.1371/journal.ppat.1008517 32365139 PMC7252634

[B178] Powers A. M. Roehrig J. T. (2011). Alphaviruses. Methods Mol. Biol. 665, 17–38. doi: 10.1007/978-1-60761-817-1_2 21116793

[B179] Priya R. Patro I. K. Parida M. M. (2014). TLR3 mediated innate immune response in mice brain following infection with Chikungunya virus. Virus Res. 189, 194–205. doi: 10.1016/j.virusres.2014.05.010 24905288

[B180] Qian Q. Zhou H. Shu T. Mu J. Fang Y. Xu J.. (2020). The capsid protein of semliki forest virus antagonizes RNA interference in mammalian cells. J. Virol. 94 (3), e01233-19. doi: 10.1128/JVI.01233-19 31694940 PMC7000971

[B181] Rathore A. P. Ng M. L. Vasudevan S. G. (2013). Differential unfolded protein response during Chikungunya and Sindbis virus infection: CHIKV nsP4 suppresses eIF2α phosphorylation. Virol. J. 10, 36. doi: 10.1186/1743-422X-10-36 23356742 PMC3605262

[B182] Reddy V. Mani R. S. Desai A. Ravi V. (2014). Correlation of plasma viral loads and presence of Chikungunya IgM antibodies with cytokine/chemokine levels during acute Chikungunya virus infection. J. Med. Virol. 86, 1393–1401. doi: 10.1002/jmv.23875 24523146

[B183] Reilly J. M. Xing W. Levicky V. Souccar S. Rogers C. Sathyavagiswaran L. (2020). Postmortem chikungunya diagnosis: A case report and literature review. Am. J. Forensic Med. Pathol. 41, 48–51. doi: 10.1097/PAF.0000000000000519 31977345

[B184] Rezza G. Chen R. Weaver S. C. (2017). O'nyong-nyong fever: a neglected mosquito-borne viral disease. Pathog. Glob Health 111, 271–275. doi: 10.1080/20477724.2017.1355431 28829253 PMC5694854

[B185] Rikkonen M. Peränen J. Kääriäinen L. (1992). Nuclear and nucleolar targeting signals of Semliki Forest virus nonstructural protein nsP2. Virology 189, 462–473. doi: 10.1016/0042-6822(92)90570-F 1386484

[B186] Rowell J. F. Griffin D. E. (2002). Contribution of T cells to mortality in neurovirulent Sindbis virus encephalomyelitis. J. Neuroimmunol 127, 106–114. doi: 10.1016/S0165-5728(02)00108-X 12044981

[B187] Rusnak J. M. Dupuy L. C. Niemuth N. A. Glenn A. M. Ward L. A. (2018). Comparison of aerosol- and percutaneous-acquired Venezuelan equine encephalitis in humans and nonhuman primates for suitability in predicting clinical efficacy under the animal rule. Comp. Med. 68, 380–395. doi: 10.30802/AALAS-CM-18-000027 30282570 PMC6200028

[B188] Ryman K. D. Klimstra W. B. (2008). Host responses to alphavirus infection. Immunol. Rev. 225, 27–45. doi: 10.1111/j.1600-065X.2008.00670.x 18837774

[B189] Ryman K. D. Klimstra W. B. Nguyen K. B. Biron C. A. Johnston R. E. (2000). Alpha/beta interferon protects adult mice from fatal Sindbis virus infection and is an important determinant of cell and tissue tropism. J. Virol. 74, 3366–3378. doi: 10.1128/JVI.74.7.3366-3378.2000 10708454 PMC111838

[B190] Samuel M. A. Wang H. Siddharthan V. Morrey J. D. Diamond M. S. (2007). Axonal transport mediates West Nile virus entry into the central nervous system and induces acute flaccid paralysis. Proc. Natl. Acad. Sci. U.S.A. 104, 17140–17145. doi: 10.1073/pnas.0705837104 17939996 PMC2040476

[B191] Santiago F. W. Halsey E. S. Siles C. Vilcarromero S. Guevara C. Silvas J. A.. (2015). Long-term arthralgia after mayaro virus infection correlates with sustained pro-inflammatory cytokine response. PloS Negl. Trop. Dis. 9, e0004104. doi: 10.1371/journal.pntd.0004104 26496497 PMC4619727

[B192] Santos F. M. Costa V. Araújo S. Sousa C. Moreira T. P. Gonçalves M. R.. (2024). Essential role of the CCL2-CCR2 axis in Mayaro virus-induced disease. J. Virol. 98, e0110223. doi: 10.1128/jvi.01102-23 38169294 PMC10805060

[B193] Santos F. M. Dias R. S. de Oliveira M. D. Costa I. Fernandes L. S. Pessoa C. R.. (2019). Animal model of arthritis and myositis induced by the Mayaro virus. PloS Negl. Trop. Dis. 13, e0007375. doi: 10.1371/journal.pntd.0007375 31050676 PMC6519846

[B194] Schäfer A. Brooke C. B. Whitmore A. C. Johnston R. E. (2011). The role of the blood-brain barrier during Venezuelan equine encephalitis virus infection. J. Virol. 85, 10682–10690. doi: 10.1128/JVI.05032-11 21849461 PMC3187510

[B195] Schilte C. Couderc T. Chretien F. Sourisseau M. Gangneux N. Guivel-Benhassine F.. (2010). Type I IFN controls chikungunya virus via its action on nonhematopoietic cells. J. Exp. Med. 207, 429–442. doi: 10.1084/jem.20090851 20123960 PMC2822618

[B196] Schlee M. Hartmann G. (2016). Discriminating self from non-self in nucleic acid sensing. Nat. Rev. Immunol. 16, 566–580. doi: 10.1038/nri.2016.78 27455396 PMC7097691

[B197] Schmaljohn A. L. McClain D. (1996). “Alphaviruses (Togaviridae) and flaviviruses (Flaviviridae),” in Medical microbiology, 4th ed. Ed. Baron S.(Galveston (TX: University of Texas Medical Branch at Galvesto). 21413253

[B198] Schoggins J. W. (2019). Interferon-stimulated genes: what do they all do? Annu. Rev. Virol. 6, 567–584. doi: 10.1146/annurev-virology-092818-015756 31283436

[B199] Schoggins J. W. Wilson S. J. Panis M. Murphy M. Y. Jones C. T. Bieniasz P.. (2011). A diverse range of gene products are effectors of the type I interferon antiviral response. Nature 472, 481–485. doi: 10.1038/nature09907 21478870 PMC3409588

[B200] Schultz D. R. Barthal J. S. Garrett G. (1977). Western equine encephalitis with rapid onset of parkinsonism. Neurology 27, 1095–1096. doi: 10.1212/WNL.27.11.1095 563006

[B201] Schwartz O. Albert M. L. (2010). Biology and pathogenesis of chikungunya virus. Nat. Rev. Microbiol. 8, 491–500. doi: 10.1038/nrmicro2368 20551973

[B202] Seymour R. L. Rossi S. L. Bergren N. A. Plante K. S. Weaver S. C. (2013). The role of innate versus adaptive immune responses in a mouse model of O'nyong-nyong virus infection. Am. J. Trop. Med. Hyg 88, 1170–1179. doi: 10.4269/ajtmh.12-0674 23568285 PMC3752819

[B203] Shah K. V. Baron S. (1965). Laboratory infection with chikungunya virus: a case report. Indian J. Med. Res. 53, 610–613. 5827506

[B204] Sharma A. Bhattacharya B. Puri R. K. Maheshwari R. K. (2008). Venezuelan equine encephalitis virus infection causes modulation of inflammatory and immune response genes in mouse brain. BMC Genomics 9, 289. doi: 10.1186/1471-2164-9-289 18558011 PMC2440554

[B205] Sharma A. Maheshwari R. K. (2009). Oligonucleotide array analysis of Toll-like receptors and associated signalling genes in Venezuelan equine encephalitis virus-infected mouse brain. J. Gen. Virol. 90, 1836–1847. doi: 10.1099/vir.0.010280-0 19369408

[B206] Sian-Hulsmann J. Riederer P. (2024). Virus-induced brain pathology and the neuroinflammation-inflammation continuum: the neurochemists view. J. Neural Transm (Vienna). doi: 10.1007/s00702-023-02723-5 PMC11608394 38261034

[B207] Simarmata D. Ng D. C. Kam Y. W. Lee B. Sum M. S. Her Z.. (2016). Early clearance of Chikungunya virus in children is associated with a strong innate immune response. Sci. Rep. 6, 26097. doi: 10.1038/srep26097 27180811 PMC4867653

[B208] Simmons J. D. White L. J. Morrison T. E. Montgomery S. A. Whitmore A. C. Johnston R. E.. (2009). Venezuelan equine encephalitis virus disrupts STAT1 signaling by distinct mechanisms independent of host shutoff. J. Virol. 83, 10571–10581. doi: 10.1128/JVI.01041-09 19656875 PMC2753124

[B209] Simon F. Parola P. Grandadam M. Fourcade S. Oliver M. Brouqui P.. (2007). Chikungunya infection: an emerging rheumatism among travelers returned from Indian Ocean islands. Report of 47 cases. Med. (Baltimore) 86, 123–137. doi: 10.1097/MD/0b013e31806010a5 17505252

[B210] Skidmore A. M. Bradfute S. B. (2023). The life cycle of the alphaviruses: From an antiviral perspective. Antiviral Res. 209, 105476. doi: 10.1016/j.antiviral.2022.105476 36436722 PMC9840710

[B211] Sourisseau M. Schilte C. Casartelli N. Trouillet C. Guivel-Benhassine F. Rudnicka D.. (2007). Characterization of reemerging chikungunya virus. PloS Pathog. 3, e89. doi: 10.1371/journal.ppat.0030089 17604450 PMC1904475

[B212] Staikowsky F. Talarmin F. Grivard P. Souab A. Schuffenecker I. Le Roux K.. (2009). Prospective study of Chikungunya virus acute infection in the Island of La Reunion during the 2005-2006 outbreak. PloS One 4, e7603. doi: 10.1371/journal.pone.0007603 19893613 PMC2764049

[B213] Stark G. R. Darnell J. E. Jr (2012). The JAK-STAT pathway at twenty. Immunity 36, 503–514. doi: 10.1016/j.immuni.2012.03.013 22520844 PMC3909993

[B214] Steele K. E. Davis K. J. Stephan K. Kell W. Vogel P. Hart M. K. (1998). Comparative neurovirulence and tissue tropism of wild-type and attenuated strains of Venezuelan equine encephalitis virus administered by aerosol in C3H/HeN and BALB/c mice. Vet. Pathol. 35, 386–397. doi: 10.1177/030098589803500508 9754544

[B215] Steer S. A. Corbett J. A. (2003). The role and regulation of COX-2 during viral infection. Viral Immunol. 16, 447–460. doi: 10.1089/088282403771926283 14733733

[B216] Strauss J. H. Strauss E. G. (1994). The alphaviruses: gene expression, replication, and evolution. Microbiol. Rev. 58, 491–562. doi: 10.1128/mr.58.3.491-562.1994 7968923 PMC372977

[B217] Suhrbier A. Jaffar-Bandjee M. C. Gasque P. (2012). Arthritogenic alphaviruses–an overview. Nat. Rev. Rheumatol 8, 420–429. doi: 10.1038/nrrheum.2012.64 22565316

[B218] Suhrbier A. La Linn M. (2003). Suppression of antiviral responses by antibody-dependent enhancement of macrophage infection. Trends Immunol. 24, 165–168. doi: 10.1016/S1471-4906(03)00065-6 12697441

[B219] Suthar M. S. Diamond M. S. Gale M. Jr. (2013). West Nile virus infection and immunity. Nat. Rev. Microbiol. 11, 115–128. doi: 10.1038/nrmicro2950 23321534

[B220] Swiecki M. Colonna M. (2015). The multifaceted biology of plasmacytoid dendritic cells. Nat. Rev. Immunol. 15, 471–485. doi: 10.1038/nri3865 26160613 PMC4808588

[B221] Tandale B. V. Sathe P. S. Arankalle V. A. Wadia R. S. Kulkarni R. Shah S. V.. (2009). Systemic involvements and fatalities during Chikungunya epidemic in India 2006. J. Clin. Virol. 46, 145–149. doi: 10.1016/j.jcv.2009.06.027 19640780

[B222] Tappe D. Pérez-Girón J. V. Just-Nübling G. Schuster G. Gómez-Medina S. Günther S.. (2016). Sustained elevated cytokine levels during recovery phase of mayaro virus infection. Emerg. Infect. Dis. 22, 750–752. doi: 10.3201/eid2204.151502 26981875 PMC4806971

[B223] Teo T. H. Chan Y. H. Lee W. W. Lum F. M. Amrun S. N. Her Z.. (2017). Fingolimod treatment abrogates chikungunya virus-induced arthralgia. Sci. Transl. Med. 9 (375), eaal1333. doi: 10.1126/scitranslmed.aal1333 28148838

[B224] Teo T. H. Her Z. Tan J. J. Lum F. M. Lee W. W. Chan Y. H.. (2015). Caribbean and la reunion chikungunya virus isolates differ in their capacity to induce proinflammatory th1 and NK cell responses and acute joint pathology. J. Virol. 89, 7955–7969. doi: 10.1128/JVI.00909-15 25995257 PMC4505608

[B225] Teo T. H. Lum F. M. Claser C. Lulla V. Lulla A. Merits A.. (2013). A pathogenic role for CD4+ T cells during Chikungunya virus infection in mice. J. Immunol. 190, 259–269. doi: 10.4049/jimmunol.1202177 23209328

[B226] Thiruvengadam K. V. Kalyanasundaram V. Rajgopal J. (1965). Clinical and pathological studies on chikungunya fever in Madras city. Indian J. Med. Res. 53, 729–744. 5830407

[B227] Torres J. R. Falleiros-Arlant L. H. Dueñas L. Pleitez-Navarrete J. Salgado D. M. Castillo J. B. (2016). Congenital and perinatal complications of chikungunya fever: a Latin American experience. Int. J. Infect. Dis. 51, 85–88. doi: 10.1016/j.ijid.2016.09.009 27619845

[B228] Torres-Ruesta A. Chee R. S. Ng L. F. P. (2021). Insights into antibody-mediated alphavirus immunity and vaccine development landscape. Microorganisms 9 (5), 899. doi: 10.3390/microorganisms9050899 33922370 PMC8145166

[B229] Touret Y. Randrianaivo H. Michault A. Schuffenecker I. Kauffmann E. Lenglet Y.. (2006). Early maternal-fetal transmission of the Chikungunya virus. Presse Med. 35, 1656–1658. doi: 10.1016/S0755-4982(06)74874-6 17086120

[B230] Trgovcich J. Aronson J. F. Johnston R. E. (1996). Fatal Sindbis virus infection of neonatal mice in the absence of encephalitis. Virology 224, 73–83. doi: 10.1006/viro.1996.0508 8862401

[B231] Trobaugh D. W. Klimstra W. B. (2017). Alphaviruses suppress host immunity by preventing myeloid cell replication and antagonizing innate immune responses. Curr. Opin. Virol. 23, 30–34. doi: 10.1016/j.coviro.2017.02.004 28288385 PMC5823529

[B232] Verma S. Kumar M. Gurjav U. Lum S. Nerurkar V. R. (2010). Reversal of West Nile virus-induced blood-brain barrier disruption and tight junction proteins degradation by matrix metalloproteinases inhibitor. Virology 397, 130–138. doi: 10.1016/j.virol.2009.10.036 19922973 PMC3102050

[B233] Verma S. Lo Y. Chapagain M. Lum S. Kumar M. Gurjav U.. (2009). West Nile virus infection modulates human brain microvascular endothelial cells tight junction proteins and cell adhesion molecules: Transmigration across the *in vitro* blood-brain barrier. Virology 385, 425–433. doi: 10.1016/j.virol.2008.11.047 19135695 PMC2684466

[B234] Vijayakumar K. P. Nair Anish T. S. George B. Lawrence T. Muthukkutty S. C. Ramachandran R. (2011). Clinical profile of chikungunya patients during the epidemic of 2007 in kerala, India. J. Glob Infect. Dis. 3, 221–226. doi: 10.4103/0974-777X.83526 21887052 PMC3162807

[B235] Vogel P. Abplanalp D. Kell W. Ibrahim M. S. Downs M. B. Pratt W. D.. (1996). Venezuelan equine encephalitis in BALB/c mice: kinetic analysis of central nervous system infection following aerosol or subcutaneous inoculation. Arch. Pathol. Lab. Med. 120, 164–172. 8712896

[B236] Vogel P. Kell W. M. Fritz D. L. Parker M. D. Schoepp R. J. (2005). Early events in the pathogenesis of eastern equine encephalitis virus in mice. Am. J. Pathol. 166, 159–171. doi: 10.1016/S0002-9440(10)62241-9 15632009 PMC1602312

[B237] Wang T. Town T. Alexopoulou L. Anderson J. F. Fikrig E. Flavell R. A. (2004). Toll-like receptor 3 mediates West Nile virus entry into the brain causing lethal encephalitis. Nat. Med. 10, 1366–1373. doi: 10.1038/nm1140 15558055

[B238] Ware B. C. Parks M. G. Morrison T. E. (2023). Chikungunya virus infection disrupts MHC-I antigen presentation via nonstructural protein 2. bioRxiv. 20, e1011794. doi: 10.1101/2023.11.03.565436 PMC10965081 38483968

[B239] Watts D. M. Callahan J. Rossi C. Oberste M. S. Roehrig J. T. Wooster M. T.. (1998). Venezuelan equine encephalitis febrile cases among humans in the Peruvian Amazon River region. Am. J. Trop. Med. Hyg 58, 35–40. doi: 10.4269/ajtmh.1998.58.35 9452289

[B240] Wauquier N. Becquart P. Nkoghe D. Padilla C. Ndjoyi-Mbiguino A. Leroy E. M. (2011). The acute phase of Chikungunya virus infection in humans is associated with strong innate immunity and T CD8 cell activation. J. Infect. Dis. 204, 115–123. doi: 10.1093/infdis/jiq006 21628665 PMC3307152

[B241] Weaver S. C. Osorio J. E. Livengood J. A. Chen R. Stinchcomb D. T. (2012). Chikungunya virus and prospects for a vaccine. Expert Rev. Vaccines 11, 1087–1101. doi: 10.1586/erv.12.84 23151166 PMC3562718

[B242] Webb L. G. Veloz J. Pintado-Silva J. Zhu T. Rangel M. V. Mutetwa T.. (2020). Chikungunya virus antagonizes cGAS-STING mediated type-I interferon responses by degrading cGAS. PloS Pathog. 16, e1008999. doi: 10.1371/journal.ppat.1008999 33057424 PMC7591055

[B243] Webster B. Werneke S. W. Zafirova B. This S. Coleon S. Decembre E.. (2018). Plasmacytoid dendritic cells control dengue and Chikungunya virus infections via IRF7-regulated interferon responses. Elife 7, e34273. doi: 10.7554/eLife.34273 29914621 PMC6008049

[B244] White L. K. Sali T. Alvarado D. Gatti E. Pierre P. Streblow D.. (2011). Chikungunya virus induces IPS-1-dependent innate immune activation and protein kinase R-independent translational shutoff. J. Virol. 85, 606–620. doi: 10.1128/JVI.00767-10 20962078 PMC3014158

[B245] Willems W. R. Kaluza G. Boschek C. B. Bauer H. Hager H. Schütz H. J.. (1979). Semliki forest virus: cause of a fatal case of human encephalitis. Science 203, 1127–1129. doi: 10.1126/science.424742 424742

[B246] Williamson L. E. Gilliland T. Jr. Yadav P. K. Binshtein E. Bombardi R. Kose N.. (2020). Human antibodies protect against aerosolized eastern equine encephalitis virus infection. Cell 183, 1884–1900.e1823. doi: 10.1016/j.cell.2020.11.011 33301709 PMC7806206

[B247] Winkler E. S. Shrihari S. Hykes B. L. Jr. Handley S. A. Andhey P. S. Huang Y. S.. (2020). The intestinal microbiome restricts alphavirus infection and dissemination through a bile acid-type I IFN signaling axis. Cell 182, 901–918.e918. doi: 10.1016/j.cell.2020.06.029 32668198 PMC7483520

[B248] Wongchitrat P. Chanmee T. Govitrapong P. (2024). Molecular mechanisms associated with neurodegeneration of neurotropic viral infection. Mol. Neurobiol. 61, 2881–2903. doi: 10.1007/s12035-023-03761-6 37946006 PMC11043213

[B249] Yin P. Davenport B. J. Wan J. J. Kim A. S. Diamond M. S. Ware B. C.. (2023). Chikungunya virus cell-to-cell transmission is mediated by intercellular extensions *in vitro* and *in vivo* . Nat. Microbiol. 8, 1653–1667. doi: 10.1038/s41564-023-01449-0 37591996 PMC10956380

[B250] Yin J. Gardner C. L. Burke C. W. Ryman K. D. Klimstra W. B. (2009). Similarities and differences in antagonism of neuron alpha/beta interferon responses by Venezuelan equine encephalitis and Sindbis alphaviruses. J. Virol. 83, 10036–10047. doi: 10.1128/JVI.01209-09 19641001 PMC2748036

[B251] Yu X. Lin S. G. Huang X. R. Bacher M. Leng L. Bucala R.. (2007). Macrophage migration inhibitory factor induces MMP-9 expression in macrophages via the MEK-ERK MAP kinase pathway. J. Interferon Cytokine Res. 27, 103–109. doi: 10.1089/jir.2006.0054 17316137

[B252] Zacks M. A. Paessler S. (2010). Encephalitic alphaviruses. Vet. Microbiol. 140, 281–286. doi: 10.1016/j.vetmic.2009.08.023 19775836 PMC2814892

[B253] Zaid A. Burt F. J. Liu X. Poo Y. S. Zandi K. Suhrbier A.. (2021). Arthritogenic alphaviruses: epidemiological and clinical perspective on emerging arboviruses. Lancet Infect. Dis. 21, e123–e133. doi: 10.1016/S1473-3099(20)30491-6 33160445

[B254] Ziegler S. A. Lu L. da Rosa A. P. Xiao S. Y. Tesh R. B. (2008). An animal model for studying the pathogenesis of chikungunya virus infection. Am. J. Trop. Med. Hyg 79, 133–139. doi: 10.4269/ajtmh.2008.79.133 18606777

